# Fbxo2 mediates clearance of damaged lysosomes and modifies neurodegeneration in the Niemann-Pick C brain

**DOI:** 10.1172/jci.insight.136676

**Published:** 2020-10-15

**Authors:** Elaine A. Liu, Mark L. Schultz, Chisaki Mochida, Chan Chung, Henry L. Paulson, Andrew P. Lieberman

**Affiliations:** 1 Department of Pathology,; 2 Cellular and Molecular Biology Graduate Program, and; 3 Medical Scientist Training Program, University of Michigan Medical School, Ann Arbor, Michigan, USA.; 4 Yamaguchi University School of Medicine, Ube, Yamaguchi, Japan.; 5 Department of Neurology, University of Michigan Medical School, Ann Arbor, Michigan, USA.

**Keywords:** Neuroscience, Autophagy, Lysosomes, Neurodegeneration

## Abstract

A critical response to lysosomal membrane permeabilization (LMP) is the clearance of damaged lysosomes through a selective form of macroautophagy known as lysophagy. Although regulators of this process are emerging, whether organ- and cell-specific components contribute to the control of lysophagy remains incompletely understood. Here, we examined LMP and lysophagy in Niemann-Pick type C (NPC) disease, an autosomal recessive disorder characterized by the accumulation of unesterified cholesterol within late endosomes and lysosomes, leading to neurodegeneration and early death. We demonstrated that NPC human fibroblasts show enhanced sensitivity to lysosomal damage as a consequence of lipid storage. Moreover, we described a role for the glycan-binding F-box protein 2 (Fbxo2) in CNS lysophagy. Fbxo2 functions as a component of the S phase kinase-associated protein 1–cullin 1–F-box protein (SKP1-CUL1-SCF) ubiquitin ligase complex. Loss of Fbxo2 in mouse primary cortical cultures delayed clearance of damaged lysosomes and decreased viability after lysosomal damage. Moreover, Fbxo2 deficiency in a mouse model of NPC exacerbated deficits in motor function, enhanced neurodegeneration, and reduced survival. Collectively, our data identified a role for Fbxo2 in CNS lysophagy and establish its functional importance in NPC.

## Abstract 2

Glycan binding protein Fbxo2 regulates lysophagy in the brain, and its deficiency exacerbates neuronal deficits in a mouse model of Niemann-Pick type C disease.

## Graphical abstract

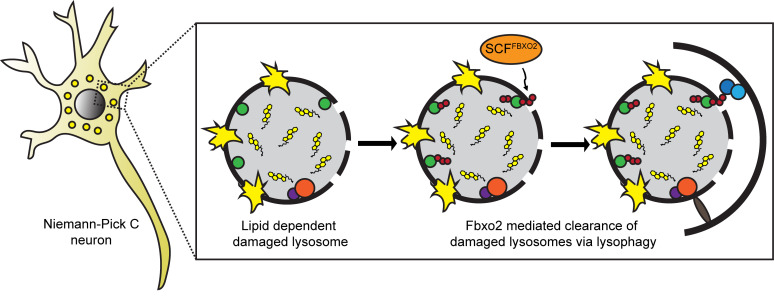

## Introduction

Lysosomes are critical organelles that function in degrading and recycling cellular waste and play broader roles in signaling, membrane repair, and metabolism (1). Because lysosomes contain diverse hydrolytic enzymes, lysosomal membrane integrity is essential for organelle function and for containing enzymes within the lysosomal compartment. A variety of factors, including lysosomotropic drugs and oxidative stress, lead to lysosomal membrane permeabilization (LMP), releasing lysosomal enzymes and triggering cellular processes from inflammasome activation to apoptosis (2). LMP-induced cell death can occur under physiologic conditions as a homeostatic response, such as during mammary gland involution or to maintain neutrophil numbers during inflammation (3). However, LMP is also observed in several neurodegenerative diseases, including Alzheimer and Parkinson diseases and lysosomal storage disorders (LSDs) (3).

One protective measure against LMP is the clearance of damaged lysosomes through a selective form of macroautophagy known as lysophagy. In this process, damaged lysosomes are sensed, and subsequent ubiquitination of lysosomal proteins leads to recruitment of autophagic machinery, engulfment by autophagic membranes, and clearance of the damaged organelles (2). Cytosolic galectins (Gals), including Gal1, -3, -8, and -9, serve as sensors of lysosomal damage (4–7). In addition to their sensing function, Gals also play more active roles in lysophagy by recruiting autophagy adapters; Gal3 interacts with TRIM16 (5), and Gal8 recruits NDP52 (7). A key intermediate step for lysophagy progression is ubiquitination of lysosomal proteins. Polyubiquitination of organelle membrane proteins is a feature of many forms of selective autophagy, which mediates recruitment of autophagic machinery, allowing for efficient organelle turnover (2, 8). LRSAM1 (9), TRIM16 (5), and the SCF^FBXO27^ ubiquitin ligase complex (10) are ubiquitin ligases that are implicated in lysophagy. Although components of lysophagy are identified in recent studies, aspects of the machinery that function in organ and cell type–specific regulation remain incompletely understood. Notably, LMP is observed in an increasing number of neurodegenerative diseases, yet brain-specific lysophagy machinery remains unknown.

Here, we probe LMP and lysophagy in Niemann-Pick type C (NPC), an autosomal recessive LSD characterized by accumulation of unesterified cholesterol in lysosomes and late endosomes (11). LSDs are a heterogeneous group of more than 70 inherited disorders characterized by the accumulation of lysosomal substrates due to organellar dysfunction and frequently cause neurodegeneration (12). LMP is implicated in an increasing number of lysosomal diseases, including Gaucher disease (13), late infantile neuronal ceroid lipofuscinosis (14), Niemann-Pick type A (15–17), and NPC (18, 19).

NPC is a devastating illness that often begins with liver disease, followed by a gradually worsening neurological course, with loss of motor skills, cognitive decline, seizures, and most often death by early adolescence (20). Most cases of NPC (~95%) are due to mutations in the *NPC1* gene (21), although a small subset (~5%) is due to mutations in *NPC2* (22). NPC1 is a multipass transmembrane protein in the limiting membrane of lysosomes, whereas NPC2 is a soluble protein in the lysosomal lumen. NPC1 and NPC2 function in concert to export cholesterol from lysosomes (23); thus, mutations in either of these proteins lead to cholesterol accumulation. Although lipid accumulation is a hallmark of disease, the pathogenesis of neurodegeneration in NPC remains incompletely understood. Prior studies demonstrate mislocalization of lysosomal cathepsins outside of the lysosomal compartment in neurons in the NPC mouse brain, suggestive of LMP (18, 19). In addition, NPC1-deficient cells experience increased toxicity with oxidative stress, a known inducer of LMP, and NPC mice deficient in cystatin B, an inhibitor of cathepsins, exhibit exacerbated cerebellar degeneration (19).

Here, we sought to further establish the role of LMP in NPC pathogenesis and define mechanisms of lysophagy, a critical response to lysosomal damage. We show that NPC human fibroblasts exhibited increased lysosomal damage after exposure to lysosome damaging agents and that this sensitivity was dependent upon the presence of stored lipids. Furthermore, we describe a role for F-box protein 2 (Fbxo2) in CNS lysophagy. Fbxo2 is a glycan-binding Fbxo protein that functions in the S phase kinase-associated protein 1–cullin 1–F-box protein (SKP1-CUL1-SCF) ubiquitin ligase complex, one of the largest classes of E3 ubiquitin protein ligases. We demonstrate that Fbxo2 localized to damaged lysosomes and that Fbxo2 deficiency impaired clearance of damaged lysosomes and exacerbated the NPC disease phenotype.

## Results

### I1061T NPC1 human fibroblasts are more sensitive to lysosomal damage.

**Figure 1:**
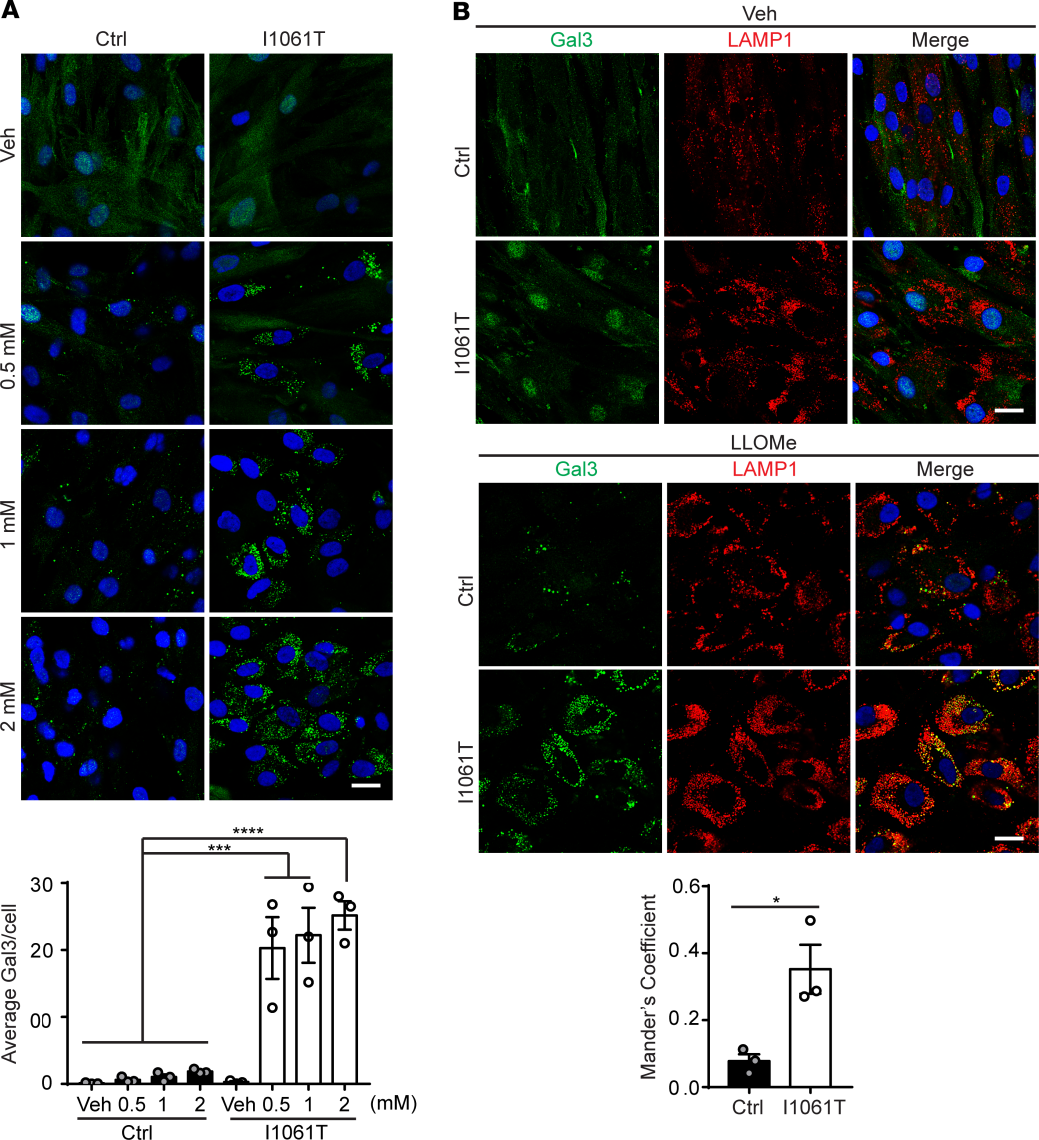
I1061T NPC1 human fibroblasts are more sensitive to lysosomal damage. (**A**) Primary human fibroblasts homozygous for WT NPC1 (Ctrl) or I1061T NPC1 (I1061T) were treated with Veh or indicated doses of LLOMe for 1 hour and stained for Gal3 to detect damaged lysosomes. Gal3 puncta per cell quantified below. (**B**) Ctrl and I1061T human fibroblasts were treated with Veh or 2 mM LLOMe for 1 hour and stained for Gal3 and LAMP-1. Colocalization was performed on 4 fields each from 3 independent experiments, with 100–200 cells per experiment. Data are shown as mean ± SEM from 3 independent experiments. *P ≤ 0.05, ***P ≤ 0.001, ****P ≤ 0.0001 by (**A**) 1-way ANOVA with Tukey’s multiple comparisons (**B**) *t* test [(**A**) *F* = 24.04, (**B**) *F* = 12.78]. Scale bar: 25 μm. NPC, Niemann-Pick disease type C; LLOMe, L-leucyl-L-leucine methyl ester; Ctrl, control; Gal3, galectin-3; Veh, vehicle.

Prior work describes LMP in Purkinje neurons and cerebellar lysates of NPC mice, as evidenced by cytosolic mislocalization of cathepsins outside of the lysosomal compartment (18, 19). To further investigate the role of LMP in NPC disease pathogenesis, we used control (Ctrl) fibroblasts homozygous for WT NPC1 and NPC human fibroblasts homozygous for I1061T NPC1 (I1061T), the most common disease-causing allele in patients of Western European ancestry (24). Cells were treated with increasing doses of the lysosomotropic compound l-leucyl-l-leucine methyl ester (LLOMe), a widely used lysosomal damaging agent that accumulates in lysosomes and is converted to a membranolytic form, (Leu-Leu)n-OME (*n* > 3), by a lysosomal thiol protease, dipeptidyl peptidase I (4, 25, 26). To detect damaged lysosomes, we quantified the number of Gal3 puncta, which accumulate on damaged lysosomes and are a sensitive indicator of LMP (4, 6). Even at the lowest doses of LLOMe, I1061T human fibroblasts exhibited significantly higher levels of Gal3 puncta per cell (Figure 1A). Because accumulated storage of lysosomal substrates leads to an increase in both size and number of lysosomes (27), we wondered if the greater number of damaged lysosomes also reflected a greater proportion of damaged lysosomes in NPC cells. To address this, we treated Ctrl and I1061T human fibroblasts with LLOMe, stained for both Gal3 and LAMP-1, and quantified their colocalization (Figure 1B). A significantly higher portion of LAMP-1 signal colocalized with Gal3 in I1061T human fibroblasts (~40%) compared with Ctrl (~10%), indicating a higher percentage of damaged lysosomes.

**Figure 2:**
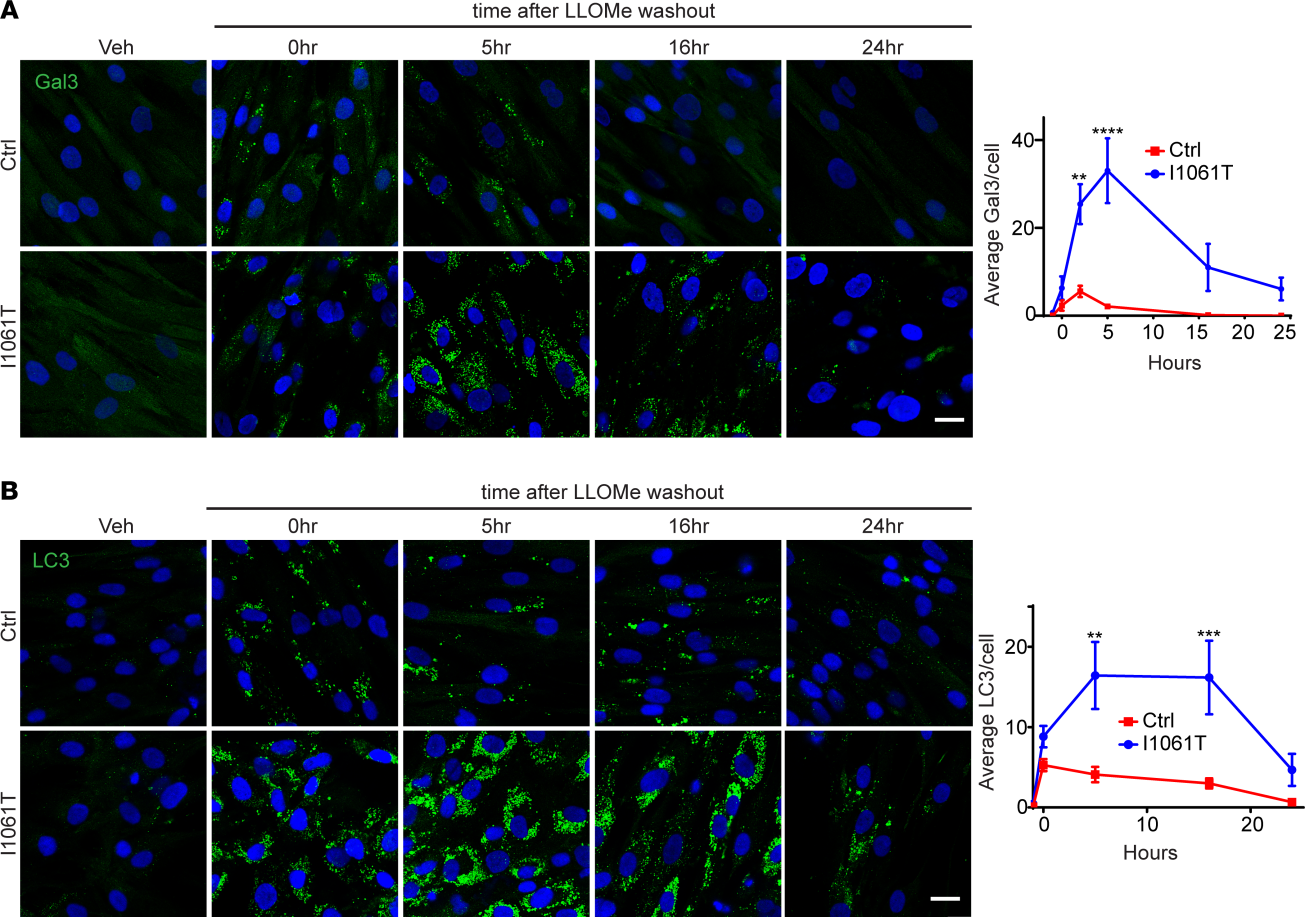
Gal3 and LC3 puncta induced by lysosomal damage are cleared in I1061T NPC1 human fibroblasts. (**A**) Ctrl and I1061T human fibroblasts were treated with Veh or 2 mM LLOMe for 1 hour and stained for Gal3 at indicated times after washout. Quantified at the right. (**B**) Ctrl and I1061T human fibroblasts were treated with Veh or 0.5 mM LLOMe for 1 hour and stained for LC3 at indicated times. LC3 puncta per cell quantified at the right. Data are shown as mean ± SEM from (**A**) 3 or (**B**) 4 independent experiments. **P ≤ 0.01, ***P ≤ 0.001, ****P ≤ 0.0001 by 2-way ANOVA with Holm-Šidák’s test. Scale bar: 25 μm. Gal3, galectin-3; NPC, Niemann-Pick disease type C; Ctrl, control; Veh, vehicle; LLOMe, L-leucyl-L-leucine methyl ester.

To limit cytotoxic consequences of LMP, damaged lysosomes are eliminated by a form of selective macroautophagy known as lysophagy (5, 7, 8, 10, 25, 28). Because impairments in autophagic flux are characterized in NPC (29–35), we wondered whether the increased lysosomal damage was due to deficient autophagic clearance. Thus, we performed a time course, treating Ctrl and I1061T human fibroblasts with LLOMe for 1 hour followed by washout, and Gal3 puncta were quantified up to 24 hours. Cells that are deficient in autophagy exhibit impaired clearance of Gal3 puncta (25, 28). Our time course confirmed significantly increased Gal3 puncta per cell in I1061T human fibroblasts but showed that these puncta were cleared through time, decreasing markedly by 16 and 24 hours (Figure 2A) and indicating functioning lysophagy. To assess if markers of autophagy also correlated with the transient accumulation and subsequent clearance of Gal3 puncta, we quantified LC3 puncta after LLOMe treatment. Similar to the pattern seen with Gal3 (Figure 2A), I1061T human fibroblasts exhibited significantly higher numbers of LC3 puncta per cell compared with Ctrl, and these puncta were cleared out by 24 hours (Figure 2B). Plotting the percentage of Gal3 and LC3 puncta clearance through time also showed that both Ctrl and I1061T human fibroblasts clear Gal3 and LC3 puncta at similar rates (Supplemental Figure 1; supplemental material available online with this article; https://doi.org/10.1172/jci.insight.136676DS1). These data support the induction of lysophagy and clearance of damaged lysosomes after treatment with LLOMe.

### Increased lysosomal damage is dependent upon lipid storage.

**Figure 3:**
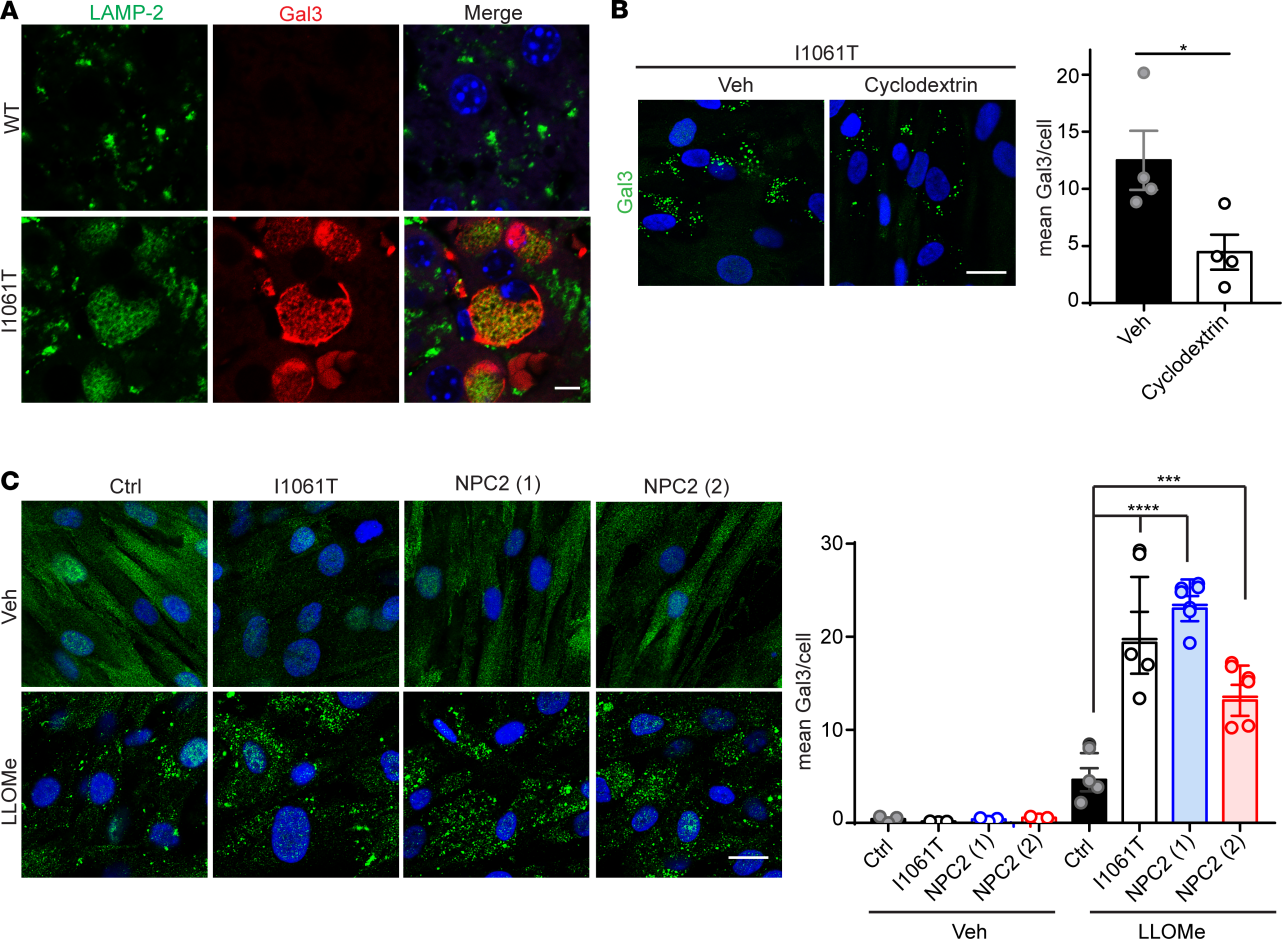
Increased lysosomal damage is dependent upon lipid storage. (**A**) Seven-week-old WT and I1061T mice were treated with vinblastine for 2 hours. Liver was collected and stained for Gal3 and LAMP-2. Mander’s coefficient in I1061T liver: 0.76. Scale bar: 5 μm. (**B**) Ctrl and I1061T human fibroblasts were treated with Veh or 1 mM cyclodextrin (Cyclo) for 48 hours and then treated with 2 mM LLOMe for 1 hour and stained for Gal3. Quantified at the right. Scale bar: 25 μm. (**C**) Ctrl, I1061T, and 2 independent lines of NPC2 human fibroblasts were treated with Veh or 2 mM LLOMe for 1 hour and stained for Gal3. Quantified at the right. Scale bar: 25 μm. Data are shown as mean ± SEM from (**B** and **C**) 4 independent experiments. *P ≤ 0.05, ***P ≤ 0.001, ****P ≤ 0.0001 by (**B**) *t* test (**C**) 1-way ANOVA with Tukey’s multiple comparisons [(**B**) *F* = 2.845, (**C**) *F* = 30.99]. Gal3, galectin-3; Ctrl, control; Veh, vehicle; NPC, Niemann-Pick disease type C; LLOMe, L-leucyl-L-leucine methyl ester.

In addition to the marked accumulation of Gal3 puncta in NPC human fibroblasts after inducing lysosomal damage with LLOMe, we also observed lysosomal damage in Npc1-I1061T–knockin mice (36). These mice develop age-dependent phenotypes, including cholesterol accumulation, neuron loss, motor impairment, and early death. WT and mutant mice were treated with vinblastine for 2 hours to slow autophagosome maturation (37). This facilitated the identification of Gal3^+^, Lamp-2^+^ vesicles in liver macrophages of Npc1-I1061T mutants (Mander’s coefficient = 0.76 for I1061T), suggesting the occurrence of lysosomal damage in vivo (Figure 3A).

Because accumulated lipids contribute to LMP (15–17, 38–40), we reasoned that lipid storage in I1061T human fibroblasts could increase sensitivity to lysosomal damage. To test this notion, we treated I1061T human fibroblasts with hydroxypropyl-β-cyclodextrin to remove stored lipids including unesterified cholesterol. Treatment with cyclodextrin for 48 hours did not significantly change levels of LAMP-1 (Supplemental Figure 2), indicating that lysosome number was unaltered during the experiment. However, cyclodextrin treatment significantly reduced Gal3 puncta per cell in I1061T human fibroblasts after treatment with LLOMe, demonstrating that sensitivity to lysosomal damage was dependent upon lipid storage (Figure 3B). The mutant I1061T NPC1 protein is known to misfold in the endoplasmic reticulum and be degraded, preventing its trafficking to the lysosome (37, 41). To examine whether loss of NPC1 at the lysosomal membrane plays a role in sensitivity to lysosomal damage, we treated NPC2-mutant human fibroblasts with LLOMe. These cells express WT NPC1 yet accumulate lipids due to functional deficiency of NPC2 (42–44). Similar to I1061T human fibroblasts, 2 independent lines of NPC2-mutant fibroblasts (g.IVS1+2T>C/g.IVS1+2T>C and c.58G>T/c.140G>T) also exhibited significantly increased lysosomal damage (Figure 3C). These data corroborate our findings from NPC1-I1061T human fibroblasts that lipid accumulation is linked to lysosomal damage. Further, they support the notion that lipid accumulation, independent of loss of NPC1 at the lysosomal membrane, contributes to increased lysosomal damage.

### Increased lysosomal damage is not due to impaired clearance.

**Figure 4:**
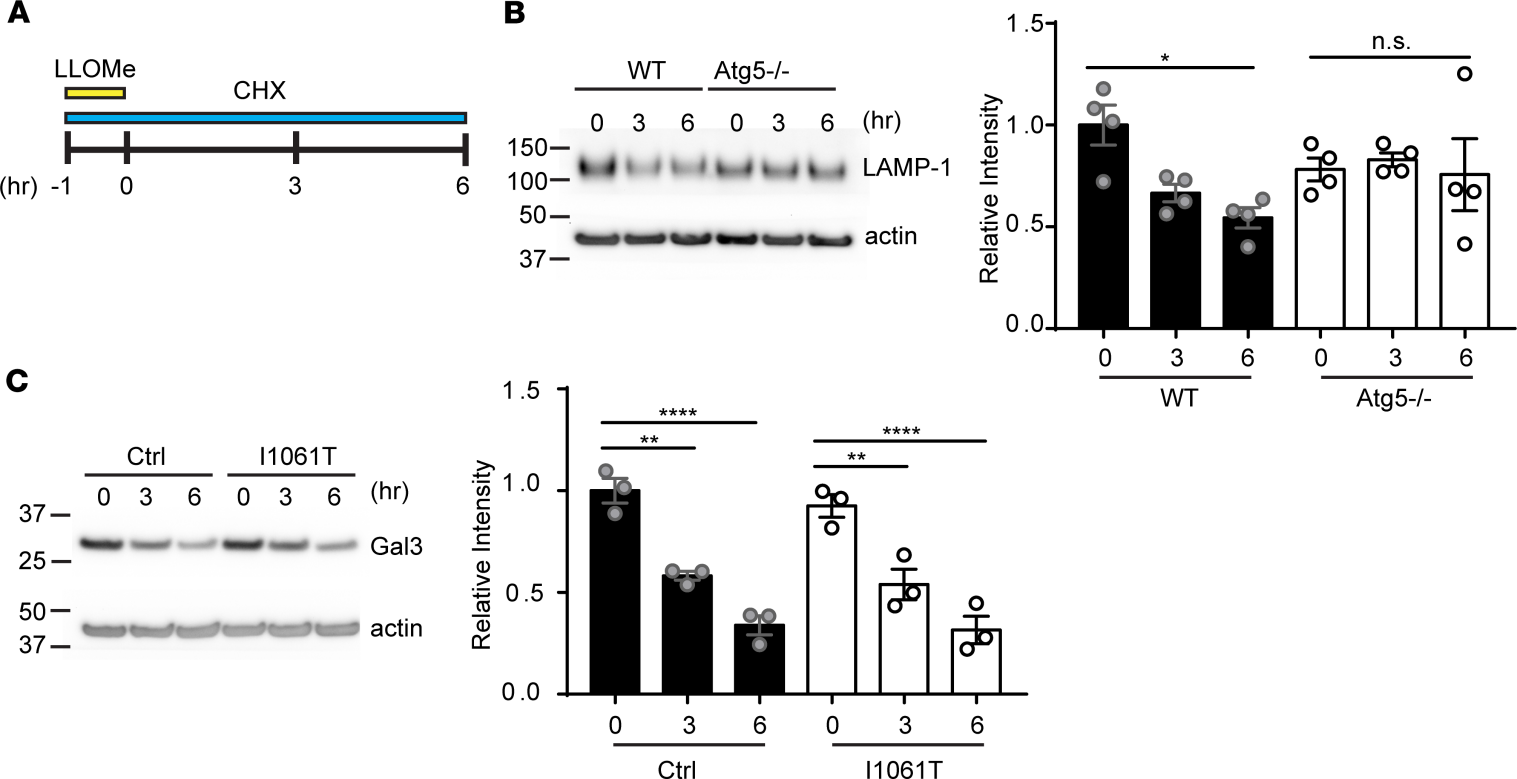
Increased lysosomal damage is not due to impaired clearance. (**A**) To examine lysophagy progression, cells were treated with 30 μg/mL CHX and 2 mM LLOMe for 1 hour and CHX treatment continued for indicated times before lysates were collected. (**B**) WT and Atg5^–/–^ MEFs were treated as in **A**. LAMP-1 levels were analyzed and quantified at the right. (**C**) Ctrl and I1061T human fibroblasts were treated as in **A**. Gal3 levels were analyzed and quantified at the right. Data are shown as mean ± SEM from (**B**) 4 or (**C**) 3 independent experiments. *P ≤ 0.05, **P ≤ 0.01, ****P ≤ 0.0001 by 1-way ANOVA with Tukey’s multiple comparisons [(**B**) *F* = 2.862, (**C**) *F* = 25.49]. CHX, cycloheximide; LLOMe, L-leucyl-L-leucine methyl ester; Ctrl, control.

Our prior time course experiments demonstrated clearance of Gal3 puncta in I1061T human fibroblasts (Figure 2A and Supplemental Figure 1), suggesting functioning lysophagy. To examine the rate at which damaged lysosomes were cleared from cells, we used a cycloheximide (CHX) chase assay (10) (Figure 4A). Cells were treated with LLOMe and CHX for 1 hour. After LLOMe washout, CHX treatment was continued for various times to assess degradation rates of LAMP-1 or Gal3 as indicators of lysosomal clearance. We first corroborated the use of this assay as a readout of lysophagy. Without lysosomal damage, LAMP-1 and Gal3 levels remained constant for the duration of the CHX chase (Supplemental Figure 3). After lysosomal damage, LAMP-1 levels diminished with time in WT mouse embryonic fibroblasts (WT MEFs), but this effect was prevented in Atg5^–/–^ MEFs, which are autophagy deficient (45) (Figure 4B). We next performed this analysis on Ctrl and I1061T human fibroblasts. Consistent with prior time course experiments (Figure 2), we found that Gal3 was cleared in both Ctrl and I1061T human fibroblasts at equivalent rates (Figure 4C) and, similarly, LAMP-1 was efficiently cleared from I1061T human fibroblasts (Supplemental Figure 4). We conclude that NPC cells exhibit enhanced sensitivity to lysosomal damage but clear damaged lysosomes at a similar rate to Ctrls.

### Fbxo2 is the most highly expressed glycan-binding F-box protein in the brain.

**Figure 5:**
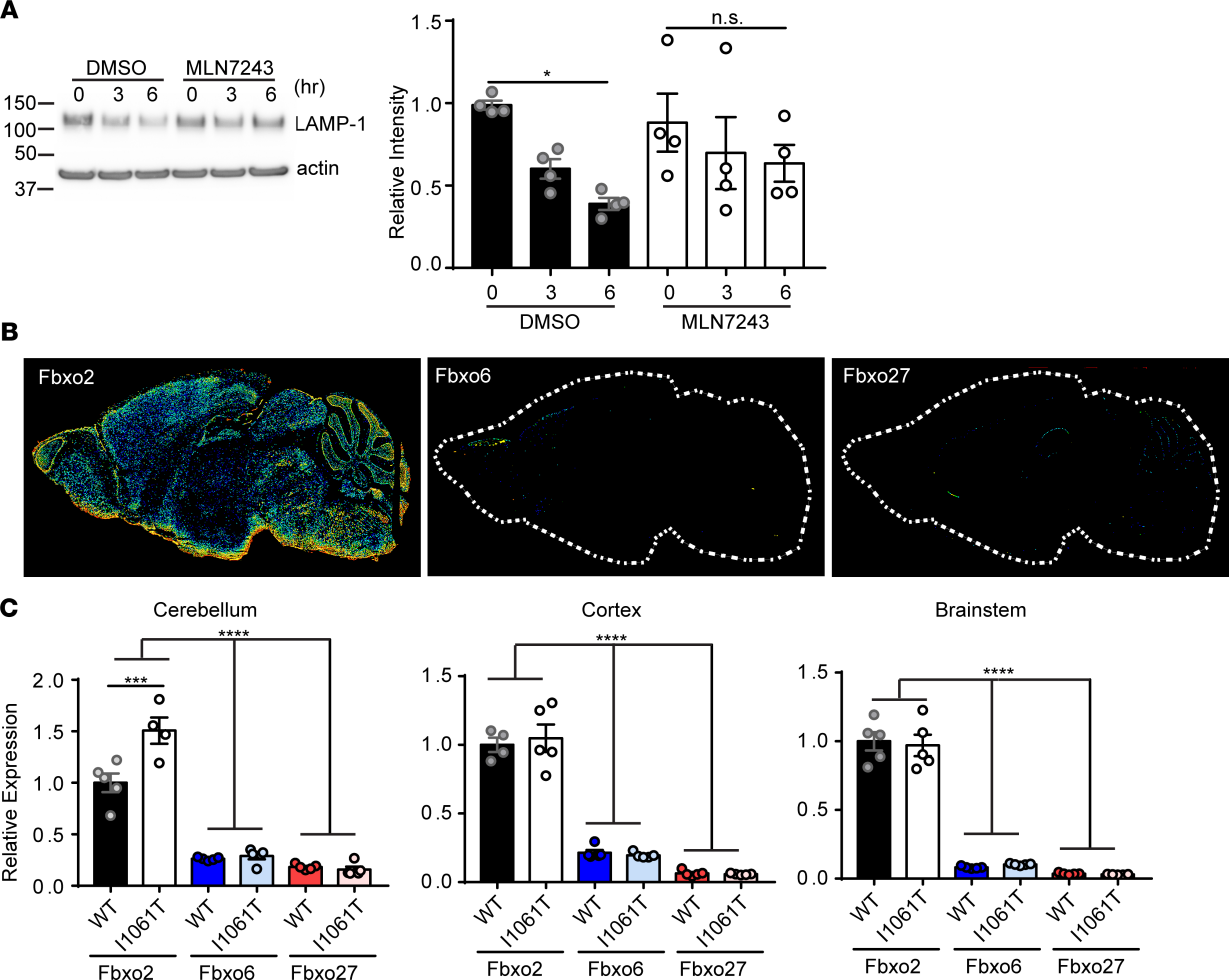
Fbxo2 is the most highly expressed glycan-binding F-box protein in the brain. (**A**) Ctrl patient fibroblasts were pretreated with DMSO or 1 μM MLN7243 for 4 hours and then treated as indicated with 30 μg/mL CHX and 2 mM LLOMe. LAMP-1 levels quantified at the right. (**B**) Allen Brain Atlas expression data of Fbxo2, Fbxo6, and Fbxo27 in mouse brain. (**C**) Relative expression of Fbxo2, Fbxo6, and Fbxo27 was determined in the cerebellum, cortex, and brainstem of WT and I1061T mice at 12 weeks by qPCR. *N* = 4–5 mice per genotype. Data are shown as mean ± SEM from (**A**) 4 independent experiments. *P ≤ 0.05, ***P ≤ 0.001, ****P ≤ 0.0001 by (**A** and **C**) 1-way ANOVA with Tukey’s multiple comparisons [(**A**) *F* = 2.803, (**C**) *F* = 78.88 (CB), *F* = 95.66 (CX), *F* = 128.7 (BS)]. Fbxo2, F-box protein 2; Ctrl, control; CHX, cycloheximide; LLOMe, L-leucyl-L-leucine methyl ester; CB, cerebellum; CX, cortex; BS, brainstem.

With evidence that NPC fibroblasts are more susceptible to lysosomal damage, we sought to further elucidate mechanisms of lysophagy, a critical cellular response to lysosomal damage. LMP exposes N-glycan–modified proteins of the limiting membrane of the lysosome to the cytosol, making them accessible to lectin binding. A recent study showed that after lysosomal damage, Fbxo27, a glycan-binding F-box protein, ubiquitinates lysosomal proteins and targets damaged lysosomes for degradation by autophagy (10). F-box proteins function within the SCF ubiquitin ligase complex, one of the largest classes of E3 ubiquitin protein ligases. There are approximately 70 different F-box proteins in humans, and the variable F-box protein determines substrate specificity (46). Confirming the importance of ubiquitination in lysophagy, inhibiting E1 ubiquitin–activating enzymes with MLN7423 (47) delayed clearance of damaged lysosomes (Figure 5A).

Prior studies provide evidence of LMP in the NPC brain (19) and establish neurons as a critical cell type for disease pathogenesis (48). Therefore, we were curious as to the function of Fbxo27 in the brain. However, using the Allen Brain Atlas we found that Fbxo27 exhibited very low expression in the brain (Figure 5B). Because the importance of ubiquitination in lysophagy is described (2), we wondered whether other glycan-binding F-box proteins might play a more significant role in the brain. Fbxo2, Fbxo6, and Fbxo27 are in the F-box associated (FBA) family of F-box proteins, which is the only family of ubiquitin ligase subunits thought to target glycoproteins (49). Using the Allen Brain Atlas we found that both Fbxo27 and Fbxo6 exhibited low brain expression; in contrast, Fbxo2 was highly expressed throughout the brain (Figure 5B). Indeed, Fbxo2 is originally identified as a brain enriched F-box protein (50), with expression annotated in neurons, astrocytes, oligodendrocytes, and microglia (51). We confirmed by quantitative PCR (qPCR) that Fbxo2 was the most highly expressed glycan-binding F-box protein in multiple brain regions, with no significant change in expression in WT compared with Npc1-I1061T–knockin mice (36), except for a slight increase in the cerebellum (Figure 5C). Similarly, protein levels of Fbxo2 were not significantly different between WT and Npc1-I1061T mice (Supplemental Figure 5).

### Fbxo2 localizes to damaged lysosomes.

**Figure 6:**
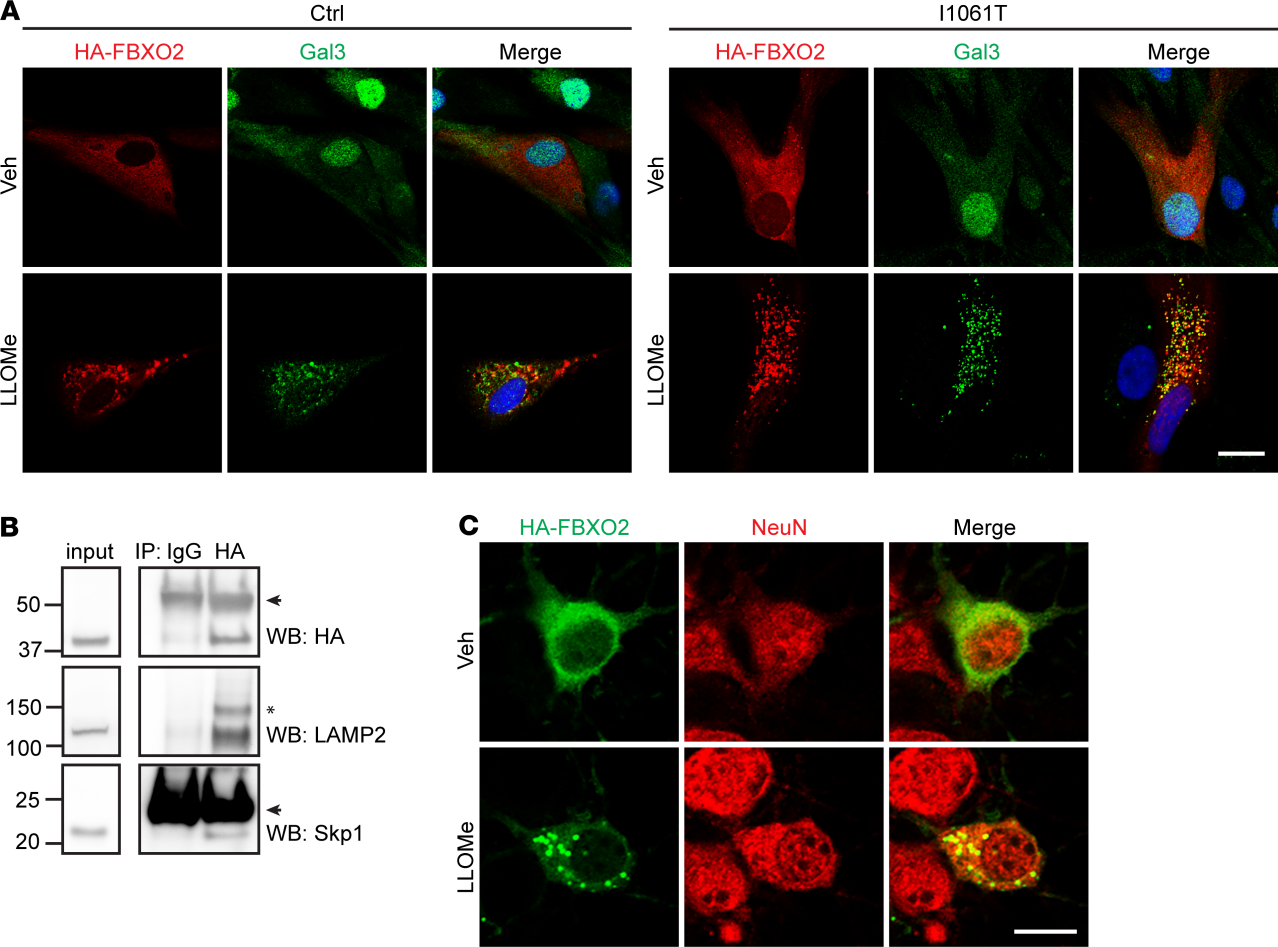
Fbxo2 localizes to damaged lysosomes. (**A**) Ctrl and I1061T human fibroblasts were transfected with HA-FBXO2, then treated with Veh or 1 mM LLOMe for 1 hour and stained for HA and Gal3. Colocalization is indicated by yellow staining in merged image. Mander’s coefficients: 0.80 (Ctrl) and 0.81 (I1061T). (**B**) I1061T human fibroblasts were electroporated with HA-FBXO2 and, after 48 hours, treated with 2 mM LLOMe for 2 hours. Lysates were immunoprecipitated with either HA antibody or Ctrl IgG. Arrowheads at approximately 50kD and approximately 25kD indicate immunoglobulin heavy and light chains, respectively. Asterisk denotes a nonspecific band detected by the LAMP2 antibody. (**C**) WT primary cortical cultures were transfected with HA-FBXO2 and treated with Veh or 2 mM LLOMe for 1 hour on D9IV. Cell were stained for HA and NeuN. Fbxo2, F-box protein 2; Ctrl, control; Veh, vehicle; LLOMe, L-leucyl-L-leucine methyl ester; Gal3, galectin-3.

Fbxo2 plays a role in glycoprotein quality control through ER-associated degradation (52). To our knowledge, it has not been shown to play a role in lysophagy, though prior work demonstrates interaction with LAMP-1 and LAMP-2 after lysosomal damage (10). To begin to investigate whether Fbxo2 contributes to lysophagy, we overexpressed HA-FBXO2 in Ctrl and I1061T human fibroblasts. As anticipated, this manipulation did not affect cholesterol accumulation in I1061T fibroblasts (Supplemental Figure 6). Notably, we found a strikingly altered distribution of HA-FBXO2 after lysosomal damage. Prior to LLOMe treatment, HA-FBXO2 exhibited diffuse, cytoplasmic staining, similar to Gal3 (Figure 6A). After lysosomal damage, HA-FBXO2 became punctate and partially colocalized with Gal3, indicating recruitment to damaged lysosomes (Mander’s coefficient = 0.80 [Ctrl], 0.81 [I1061T]) (Figure 6A). This recruitment of HA-FBXO2 to damaged lysosomes suggested that it may function in their clearance. Overexpressed FBXO6 and FBXO27 showed similar recruitment to Gal3 puncta after lysosomal damage (Supplemental Figure 7), raising the possibility that functional differences among the FBA family of F-box proteins is determined, in part, by expression patterns, with Fbxo2 expression in the CNS being most prominent. Coimmunoprecipitation experiments after LLOMe treatment confirmed that HA-FBXO2 interacted with LAMP-2 and Skp1, demonstrating its interaction with damaged lysosomes in the SCF complex (Figure 6B). We also observed interaction of HA-FBXO2 with LAMP-2 and Skp1 after vehicle treatment (Supplemental Figure 8), which is consistent with prior coimmunoprecipitation experiments with FBXO2, FBXO6, and FBXO27 before and after lysosomal damage (10) and suggests their recruitment to damaged lysosomes even in the absence of treatment with a lysosomal damaging agent. With evidence that HA-FBXO2 is recruited to damaged lysosomes in human fibroblasts, we asked whether Fbxo2 plays a role in lysophagy in the CNS. In primary cortical cultures transfected with HA-FBXO2, we saw a similar transition from diffuse cytoplasmic to punctate staining in neurons after lysosomal damage (Figure 6C) and partial colocalization with LAMP-1 (Supplemental Figure 9), indicating recruitment to damaged lysosomes.

### Fbxo2 plays a role in CNS lysophagy.

**Figure 7:**
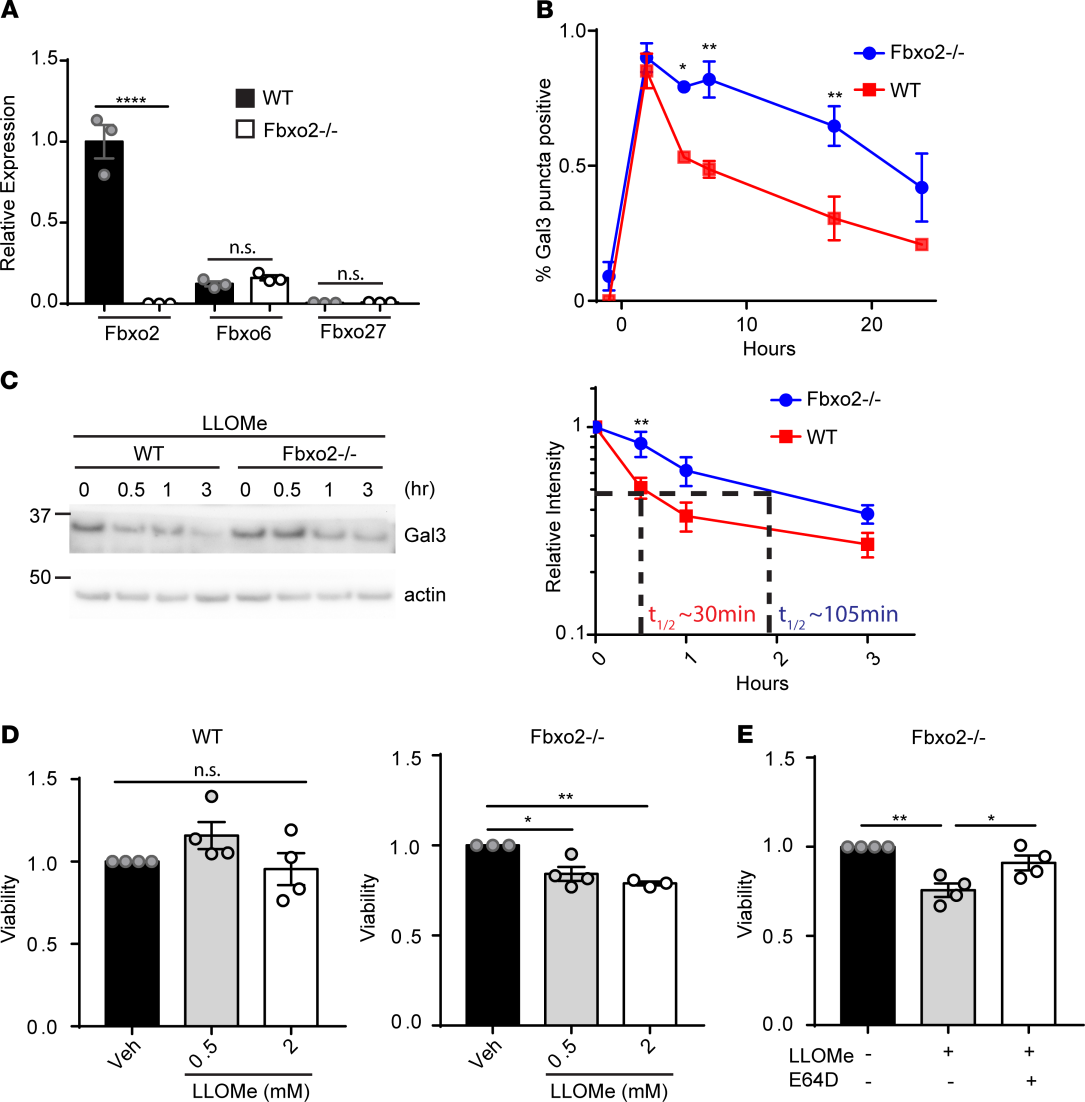
Fbxo2 mediates CNS lysophagy. (**A**) Relative expression of Fbxo2, Fbxo6, and Fbxo27 was determined in WT and Fbxo2^–/–^ primary cortical cultures at D9IV by qPCR. (**B**) WT and Fbxo2^–/–^ primary cortical cultures were transfected with EGFP-hGal3 on D4IV, treated with Veh or 2 mM LLOMe for 1 hour, and stained at various times after washout. Percentage of cells with Gal3+ puncta was quantified. (**C**) WT and Fbxo2^–/–^ primary cortical cultures were treated on D9IV with 30 μg/mL CHX and 2 mM LLOMe for 1 hour, and CHX treatment continued for indicated times before lysates were collected. Gal3 levels quantified at the right. (**D**) WT and Fbxo2^–/–^ primary cortical cultures were treated with Veh or LLOMe (0.5 mM or 2 mM) for 1 hour, and cell viability was determined by XTT assay 4 hours after LLOMe washout. (**E**) Fbxo2^–/–^ primary cortical cultures were pretreated with Veh (-) or 100 μM E64D for 30 minutes and then treated with 2 mM LLOMe for 1 hour, and cell viability was determined by XTT assay 4 hours after LLOMe washout. Data are shown as mean ± SEM from (**A**, **B**, and **D**) 3, (**C**) 5, or (**E**) 4 independent experiments. *P ≤ 0.05, **P ≤ 0.01, ****P ≤ 0.0001 by (**A** and **D**–**E**) 1-way ANOVA with Tukey’s multiple comparisons or (**B** and **C**) 2-way ANOVA with Holm-Šidák’s test [(**A**) *F* = 81.08, (**D**) *F* = 23.2, (**E**) *F* = 14.55]. Fbxo2, F-box protein 2; Veh, vehicle; LLOMe, L-leucyl-L-leucine methyl ester; Gal3, galectin-3.

To investigate a role for Fbxo2 in lysophagy in the brain, we established primary cortical cultures from WT and Fbxo2-deficient (Fbxo2^–/–^) mice (53). qPCR demonstrated that Fbxo2 was the most highly expressed glycan-binding F-box protein in WT primary cortical cultures and that there was no compensatory upregulation of Fbxo6 or Fbxo27 in response to Fbxo2 deficiency (Figure 7A). Notably, clearance of damaged lysosomes was significantly delayed in Fbox2^–/–^ cultures. When we overexpressed Gal3 in WT and Fbxo2^–/–^ primary cortical cultures, we found significantly slower clearance of Gal3 puncta in Fbxo2^–/–^ cultures (Figure 7B). Further, by CHX chase assay (Figure 4A), the half-life of Gal3 was extended from approximately 30 minutes in WT cultures to approximately 105 minutes in Fbxo2^–/–^ primary cortical cultures (Figure 7C). These data demonstrate delayed lysophagy progression and support a role for Fbxo2 in the clearance of damaged lysosomes. Because lysosomal membrane damage releases luminal enzymes and can lead to cell death (2), we next compared cell viability in WT and Fbxo2^–/–^ primary cortical cultures after lysosomal damage. When assessing toxicity 4 hours after LLOMe treatment, we observed significantly decreased viability in Fbxo2^–/–^ cultures compared with WT (Figure 7D), indicating increased cell death after lysosomal damage. Supporting on-target effects of LLOMe, this toxicity was rescued by preventing lysosomal damage with the cathepsin inhibitor E64D (54) (Figure 7E and Supplemental Figure 10).

### Loss of Fbxo2 exacerbates the NPC disease phenotype.

**Figure 8:**
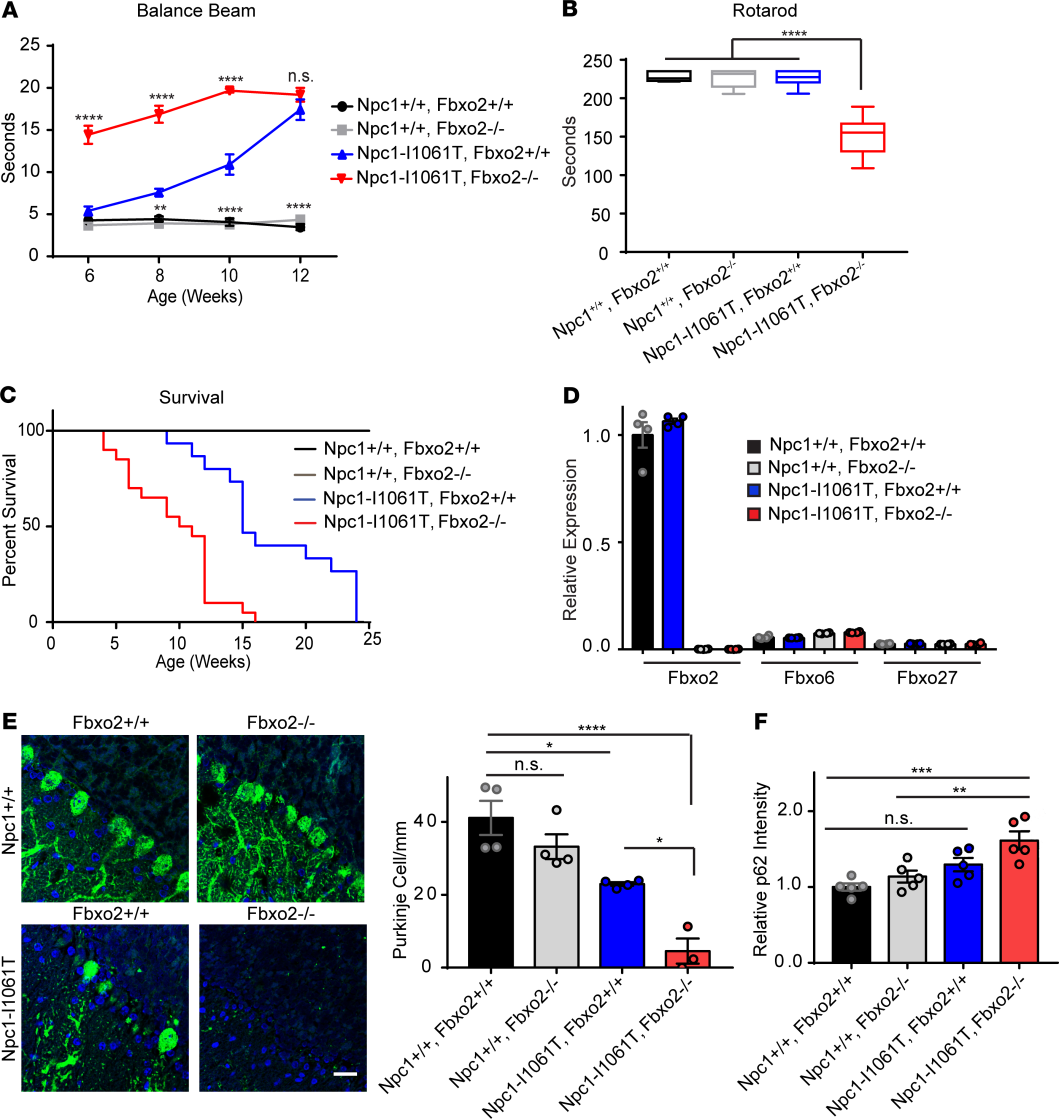
Loss of Fbxo2 exacerbates the NPC disease phenotype. (**A**) Age-dependent performance on balance beam. Mice were trained at 5 weeks and run every other week starting at 6 weeks. The average of 3 trials was taken and max time was set at 20 seconds. *N* = 5 males and 5 females per genotype. (**B**) Performance on accelerating rotarod from 4–40 rpm at 9 weeks. *N* = 5 males and 5 females per genotype. (**C**) Kaplan-Meier survival curves. *N* = 6–10 males and 6–10 females per genotype. (**D**) Relative expression of Fbxo2, Fbxo6, and Fbxo27 was determined by qPCR in 8 weeks brainstem. *N* = 4 mice per genotype. (**E**) Quantification of Purkinje cell density in lobules IV and V of midline cerebellar sections. *N* = 3–4 mice per genotype. (**F**) The relative abundance of p62 in brainstem from 8 weeks mice was determined by Western blot. *N* = 5 mice per genotype. Data are shown as mean ± SEM. *P ≤ 0.05, **P ≤ 0.01, ***P ≤ 0.001, ****P ≤ 0.0001 by (**A**) 2-way ANOVA, (**B**, **E**, and **F**) 1-way ANOVA or (**C**) log-rank (Mantal-Cox) test and Gehan-Breslow-Wilcoxon test with (**A** and **E**) Bonferroni’s or (**B** and **F**) Tukey’s multiple comparisons [(**A**) *F* = 26.88, (**B**) *F* = 63.59, (**E**) *F* = 19.49, (**F**) *F* = 11.64]. Scale bar: 25 μm. Fbxo2, F-box protein 2; NPC, Niemann-Pick disease type C.

Because our analyses indicated the occurrence of increased susceptibility to lysosomal damage in NPC1- or NPC2-deficient cells, we sought to determine whether loss of Fbxo2 modified the disease phenotype in a mouse model of NPC disease. To accomplish this, we generated Npc1-I1061T mutant mice deficient in Fbxo2. Fbxo2 deficiency is well tolerated in mice, except for the occurrence of hearing loss (53). Similarly, Fbxo2^–/–^ mice were indistinguishable from WT littermates in our analyses (Figure 8). However, loss of Fbxo2 in Npc1-I1061T mice exacerbated deficits in motor function as quantified by performance on the balance beam (Figure 8A) and rotarod (Figure 8B) and significantly decreased survival (Figure 8C). Consistent with expression analysis in primary cortical cultures, loss of Fbxo2 did not lead to compensatory upregulation of Fbxo6 or Fbxo27 in the mouse brain (Figure 8D). Fbxo2-deficient mice also had similar expression levels of the SCF component Cul1 and a slight increase in expression of Rbx1 (Supplemental Figure 11).

Exacerbation of motor phenotypes prompted us to examine changes in neuropathology. We focused our analysis on Purkinje neurons, the major output neurons of the cerebellar folia, which degenerate in NPC brain (19, 55) and whose loss is sufficient to trigger motor impairment (56). Npc1-I1061T mice deficient in Fbxo2 exhibited significantly increased Purkinje cell loss, correlating with their exacerbated motor phenotypes (Figure 8E). In contrast, Fbxo2 deficiency alone did not alter Purkinje cell density. Furthermore, brain tissue from Npc1-I1061T, Fbxo2^–/–^ mice showed enhanced accumulation of p62 protein (Figure 8F and Supplemental Figure 12A) without upregulation of p62 mRNA (Supplemental Figure 12B), similar to findings in cell culture after lysosomal damage (14). Taken together, these data indicate that loss of Fbxo2 in NPC mice exacerbated behavioral phenotypes and neurodegeneration while altering markers of autophagy.

## Discussion

In this study, we describe a role for Fbxo2 in CNS lysophagy and demonstrate the importance of lysophagy as a key compensatory pathway in NPC knockin mice. We show that NPC human fibroblasts were more sensitive to lysosomal damage by LLOMe and that this occurred in the context of functioning lysophagy (Figures 1–4). Our data suggest that the primary driver of increased sensitivity to lysosomal damage was a factor intrinsic to the lysosome that affected membrane stability. Increased oxidative stress is a contributor to LMP in NPC (19), but it is likely that additional factors also function in this context. We now show that lipid storage, independent of loss of the NPC1 protein, contributed to increased sensitivity to lysosomal damage (Figure 3). Consistent with this finding, studies demonstrate that lipids, including accumulated sphingomyelin (16, 17, 41) and cholesterol (39, 40), induce lysosomal damage. Because diverse storage materials in Gaucher disease (13) and late infantile neuronal ceroid lipofuscinosis (14) also contribute to lysosomal damage, LMP is likely the consequence of aberrant accumulations within lysosomes. In contrast, studies also describe the importance of lipids in membrane rigidity and lysosomal membrane stability (57–59). Notably, the absence of functional NPC1 protein is expected to impair the movement of luminal cholesterol into the limiting membrane of the lysosome. Defining the role of altered luminal versus lysosomal membrane lipid composition and their effects on lysosomal membrane stability will be important in furthering our understanding of lysosomal dysfunction in NPC. Additionally, because some of our studies were conducted in human fibroblasts, we acknowledge that these cells may not fully mirror events in neurons in a complex disorder like NPC disease, and further work on LMP in NPC neurons will be of importance.

Lysophagy is a critical response to LMP, and we show that of the FBA family of glycoprotein binding F-box proteins, Fbxo2 was most highly expressed in the brain (Figure 5) and was recruited to damaged lysosomes (Figure 6). Supporting its function in lysophagy, loss of Fbxo2 in primary cortical cultures delayed clearance of damaged lysosomes and leads to decreased viability after lysosomal damage (Figure 7). This finding is consistent with work on the glycan-binding F-box protein Fbxo27, where Fbxo27 deficiency slows but does not abolish lysophagy (10). Likely, additional pathways function to mediate lysophagy in the brain. TRIM16, a RING-type ubiquitin ligase, also mediates lysophagy progression (5) and may function redundantly with Fbxo2.

Supporting the function of Fbxo2 in lysophagy, NPC mice deficient in Fbxo2 exhibit exacerbated disease phenotypes, with significantly worse impairments in motor function and decreased survival (Figure 8). This correlated with an increased loss of Purkinje cells and an increase in the autophagic adapter protein p62 (Figure 8). These findings support a model wherein impairing lysophagy in a background of increased lysosomal damage exacerbates the disease phenotype. The characterization of Fbxo2 as a component of the machinery that regulates efficient lysophagy in the CNS is intriguing because it demonstrates specificity of the mediators of this process. Among CNS cell types, RNA-Seq data sets demonstrate broad expression of Fbxo2 in neurons and glia (51). Loss of functional Npc1 in mouse neurons and oligodendrocytes, but not astrocytes, contributes to NPC neuropathology (30, 48, 56, 60). As such, it is possible the Fbxo2 functions in multiple cell types to maintain efficient lysophagy and promote CNS homeostasis.

Collectively, our data describe a function for Fbxo2 in lysophagy and establish its proof-of-concept disease relevance in compensating for NPC pathophysiology. Our findings suggest that strategies aimed at targeting the lysosomal cell death pathway and enhancing lysophagy function may be protective against neurodegeneration in NPC. Furthermore, additional studies to probe the function of Fbxo2 will continue to advance our understanding of mechanisms involved in protein and organellar quality control pathways that likely contribute to the neuropathology in a diverse array of lysosomal diseases.

## Methods

### Antibodies

#### Primary antibodies.

The following primary antibodies (antigen, dilution, vendor) were used: Gal3 (1:100 [IF], 1:500 [WB], sc-20157 [discontinued], Santa Cruz Biotechnology); Gal3 (1:100, sc-23938, Santa Cruz Biotechnology); LAMP-1 (1:100 [IF], H4A3, Developmental Studies Hybridoma Bank at the University of Iowa); LAMP-2 (1:10 [IF], ABL-93, Developmental Studies Hybridoma Bank at the University of Iowa); LC3B (1:500 [IF], 1:1000 [WB], NB600-1384, Novus Biologicals); LAMP-1 (1:1000, ab24170, Abcam); Calbindin-D-28K (1:500, C2724, MilliporeSigma); HA.11 (1:500, 901501, BioLegend); NeuN (1:500, ABN90, MilliporeSigma); β-Actin (1:2000, A5441, MilliporeSigma); Vinculin (1:2000, V9131, MilliporeSigma); p62 (1:1000, P0067, MilliporeSigma); Skp1 (1:1000, 610530, BD Biosciences); LAMP2 (1:100 [WB], ab25631, Abcam); FBXO2 (1:100, sc-393873, Santa Cruz Biotechnology); and FLAG (1:500, F1804, MilliporeSigma).

#### Secondary antibodies.

The following secondary antibodies (dilution, antibody, and vendor) were used: Alexa Fluor 488 goat anti–rabbit IgG (H+L) (1:500, A11008, Invitrogen); Alexa Fluor 495 Fab’2 fragment of goat anti–mouse IgG (H+L) (1:500, A11020, Invitrogen); Alexa Fluor 488 goat anti–mouse IgG (H+L) (1:500, A11029, Invitrogen); Alexa Fluor 594 goat anti–guinea pig IgG (H+L) (1:500, A11076, Invitrogen); goat anti–mouse IgG (H+L)-HRP conjugate (1:2000, Bio-Rad 170-6516); Alexa Fluor 488 goat anti–rat IgG (H+L) (1:500, A11006, Invitrogen); and goat anti–rabbit IgG (H+L)-HRP conjugate (1:2000, Bio-Rad 170-6515).

### Reagents

The following drugs and small molecules were used: MLN7243 (CT-M7243, Chemietek); Leu-Leu methyl ester hydrobromide (L7393, MilliporeSigma); CHX (C7698, MilliporeSigma); E-64d (13533, Cayman); 2-hydroxypropyl-β-cyclodextrin (H-107, MilliporeSigma); vinblastine sulfate salt (catalog V1377, MilliporeSigma); filipin (F9765, MilliporeSigma). Plasmid encoding HA-FBXO2 was a gift from Henry Paulson (University of Michigan, Ann Arbor, Michigan, USA). Plasmids encoding FLAG-FBXO2, FLAG-FBXO6, and FLAG-FBXO27 were gifts from Yukiko Yoshida (Tokyo Metropolitan Institute of Medical Science, Tokyo, Japan). Plasmid encoding EGFP-hGal3 was from addgene (73080).

### Cells

The following cell lines were obtained from the NIGMS Human Genetic Cell Repository at the Coriell Institute for Medical Research: GM08399 (Ctrl), GM18453 (I1061T/I1061T), GM18429 (NPC2-1), and GM18455 (NPC2-2). Fibroblasts were cultured in MEM (Gibco 10370), PSG (Gibco), and 20% premium FBS (Atlanta Biologicals). WT and Atg5^–/–^ MEF cell lines RCB2710 and RCB2711 were obtained from the RIKEN BRC Cell Bank and were cultured in MEM (Gibco 10370), PSG (Gibco), and 20% premium FBS (Atlanta Biologicals).

### Mice

Npc1-I1061T mice (36) were a gift from Daniel Ory (Washington University, St. Louis, Missouri, USA) and backcrossed to C57BL/6 (≥10 generations). Fbxo2^–/–^ mice (53) were a gift from Henry Paulson (University of Michigan, Ann Arbor, Michigan, USA) and on the C57BL/6 background.

### Western blot

Cell culture media was aspirated, cells were washed once with ice cold PBS, removed with a cell scraper, and centrifuged at 1000 *g* for 5 minutes at 4°C. The cell pellet was resuspended in RIPA (Teknova) with complete protease inhibitor (11836153001, Thermo Fisher Scientific) and 0.625 mg/mL N-ethylmaleimide (E3876, Sigma-Aldrich) and sonicated. For primary cortical cultures, lysates were centrifuged at 12,000*g* for 10 minutes at 4°C and the supernatant was collected. For tissue preparation, mice were perfused with saline before tissue was collected and flash frozen in liquid nitrogen. Tissue was homogenized and sonicated in RIPA buffer. Protein concentrations were determined by DC-protein assay (Bio-Rad) and normalized. Proteins were separated on NuPAGE 4%–12% Bis-Tris Protein Gels (NP0336BOX, Thermo Fisher Scientific) and transferred to Immobilon-P PVDF (0.45-μm pore size, Merck Millipore). Immunoreactivity was detected with ECL (Thermo Fisher Scientific) or SuperSignal West Pico PLUS Chemiluminescent Substrate (Thermo Fisher Scientific) and an iBright (Thermo Fisher Scientific). Quantification was performed using Image Studio. Band intensity was normalized to the indicated loading control.

### Quantitative real-time PCR (RT-qPCR)

RNA was collected using TRIzol (Thermo Fisher Scientific) according to manufacturer’s instructions and converted to cDNA using the High Capacity Reverse Transcription kit (4368814, Applied Biosystems). RT-qPCR was performed using 10 ng cDNA, FastStart TaqMan Probe Master Mix (Roche), and gene-specific FAM-labeled TaqMan probes (Thermo Fisher Scientific) for mouse Fbxo2 (Mm00805188), Fbxo6 (Mm01257500), Fbxo27 (Mm01179110), Sqstm1 (Mm0044809), Rbx1 (Mm01705487), and Cul1 (Mm00516318). Gene expression was normalized to mouse Cpsf2-Vic (Mm00489754) multiplexed within the same well. RT-qPCR was performed using an ABI 7900HT Sequence Detection System and relative expression calculated by the 2^(-ΔΔCt) method.

### Transfection

#### Human fibroblasts.

Cells were transfected with Lipofectamine LTX with Plus Reagent (Invitrogen). Briefly, 320 ng of endotoxin-free plasmid was incubated in 43 μL opti-MEM (Gibco) and.425uL PLUS reagent for 5 minutes. Separately, 43 μL opti-MEM was incubated with 1.28 uL LTX reagent for 5 minutes. Then, the LTX mixture was added to the plasmid mixture and incubated for 30 minutes before adding dropwise to cells.

#### Mouse primary cortical cultures.

Cells were transfected on D2-4IV with Lipofectamine 2000 (Invitrogen). Briefly, cells were washed 2 times with Neurobasal-A Medium (Gibco). Endotoxin-free plasmid (200 ng) was incubated with 50 μL opti-MEM (Gibco) for 5 minutes. Separately, 50 μL opti-MEM and 1 μL Lipofectamine 2000 was incubated for 5 minutes. After 5 minutes, the Lipofectamine mixture was added to the plasmid mixture and incubated 20 minutes before adding dropwise to cells. Transfection mixture was kept on cells for 20 minutes before removing, and cells were washed 2 times in Neurobasal-A Medium (Gibco).

### Immunofluorescence staining

Cells were washed 3 times with HBSS and fixed with ice cold 100% methanol for 20 minutes at –20°C. Cells were washed 3 times with PBS and placed in 2.5 mg/mL glycine for 10 minutes at room temperature. Cells were washed 3 times with PBS, permeabilized with 0.1% Triton in PBS for 20 minutes, and then placed in blocking solution (10% goat serum, 1% BSA in PBS) for 1 hour. Cells were incubated with primary antibodies diluted in blocking solution overnight at 4°C. The next day the slides were washed 3 times with PBS and incubated with secondary antibody diluted in blocking solution for 1 hour at room temperature. Slides were washed 3 times with PBS and mounted with Vectashield plus DAPI (Vector Laboratories). For LAMP-1 staining, after glycine incubation, cells were placed in saponin-blocking solution (0.02% saponin, 5% NGS, 1% BSA) for 1 hour and then incubated with primary antibody diluted in saponin-blocking solution overnight at 4°C. Slides were washed with PBS plus 0.02% saponin and incubated with secondary antibody diluted in saponin-blocking solution, washed and mounted.

For tissue preparation, mice were perfused with saline and 4% PFA and tissue was removed and post-fixed in 4% PFA overnight at 4°C before paraffin embedding. Paraffin-embedded tissues were cut on a Reichert-Jung 2030 microtome into 5 μm sections and placed on Thermo Scientific Superfrost Plus microscope slides. Sections were adhered onto slides in an oven at 55°C–60°C for 1 hour. Samples were deparaffinized and antigen retrieval was performed by boiling in 10 mM sodium citrate (pH 6.0) for 10 minutes and incubating in hot citrate solution for an additional 20 minutes and then washed 3 times in deionized water. For staining, slides were incubated in a solution with 0.1% Triton, 10% goat serum, and 1% BSA in PBS for 20 minutes. Then, slides were placed in blocking solution (10% goat serum, 1% BSA in PBS) before incubating in primary antibody diluted in blocking solution overnight at 4°C. Slides were washed 3 times in PBS and incubated for 1 hour with secondary antibody diluted in blocking solution. Slides were then washed 3 times in PBS and mounted with Vectashield plus DAPI (Vector Laboratories).

### Filipin staining

Cells were stained following the immunofluorescence protocol described above, with the exception of being fixed in 4% PFA. After the secondary antibody incubation, cells were washed and incubated with 1 mL filipin staining solution (5% FBS plus 40 uL filipin solution [1 mg filipin plus 40 μL DMSO] in PBS) for 2 hours at room temperature. Cells were washed 3 times in PBS and slides were mounted with ProLong Gold (Thermo Fisher Scientific).

### Primary cortical cultures

Cortices were dissected using the Papain Dissociation System (Worthington) from P0-P1 WT or Fbxo2^–/–^ pups. Briefly, cortices were dissected free of meninges, placed in papain solution, and incubated at 37°C for 20 minutes. Cortices were triturated 15 times with a 10-mL pipette tip and spun at 30.9*g* for 5 minutes. Cells were resuspended and spun over a discontinuous density gradient at 22*g* for 5 minutes. Cells were then resuspended in Neurobasal-A Medium (Gibco) with B-27 Supplement (Gibco), GlutaMAX (Gibco), and Pen Strep and then counted and plated. Media was changed every 3 days.

### Cell survival

The XTT assay (ATCC) was used to assess cell survival according to manufacturer’s protocol. Briefly, 50 μL of XTT solution was added to 100 μL of cell culture media for 4 hours in a CO_2_ incubator at 37°C. Plates were read on a Synergy HTX multimode plate reader (BioTek) at 475 nm and 660 nm.

### Neon Transfection System

Cells were transfected by Neon Transfection System (Thermo Fisher Scientific) according to manufacturer’s protocol. Briefly, cells were counted and resuspended in DPBS (Gibco) along with plasmid DNA. Cell culture plates were preincubated with culture medium without antibiotics. Neon Tube was set up with 3 mL Electrolytic Buffer E2 and the 100 μL Neon Tip was used at 1200V, 40 ms, 1 pulse.

### Coimmunoprecipitation

Cells were washed in PBS and cross-linked with DSP (Thermo Fisher Scientific 22585) for 30 minutes at room temperature. Tris-HCl pH 7.5 was added to a final concentration of 20 mM and cells were incubated on a rotator for 15 minutes at 4°C. Cells were centrifuged for 5 minutes at 1000*g* and resuspended in lysis buffer (0.025 M Tris, 0.15 M NaCl, 0.001 M EDTA, 1% NP-40, 5% glycerol; pH 7.4) with complete protease inhibitor (11836153001, Thermo Fisher Scientific). Lysates were precleared with protein A/G beads for 30 minutes at 4°C. Beads were pelleted at 1000*g* for 1 minute and lysates were incubated with HA (BioLegend) antibody or Ctrl IgG on a rotator overnight at 4°C. Protein A/G beads were then added and lysates tumbled at 4°C for 1 hour. Beads were placed into spin columns (Pierce) and washed 6 times in lysis buffer. Finally, beads were boiled at 95°C for 5 minutes in 6× loading buffer and loaded on NuPAGE 4%–12% 10-well gels (Invitrogen).

### Microscopy

Confocal images were collected using a Nikon A-1 confocal with diode-based laser system. Colocalization and puncta quantification were determined using CellProfiler Analyst Software. For Gal3 puncta quantification, 100–200 cells per experiment were examined.

### Phenotype analysis

#### Balance beam.

The balance beam consists of a 5 mm wide square beam suspended at 50 cm. Mice were trained at 5 weeks of age to cross the beam and then tested every other week starting at 6 weeks. For testing, mice were run 3 times across the beam, and the average time was taken. Maximum time was set at 20 seconds and falls were scored as 20 seconds.

#### Rotarod.

After acclimating to the testing room for 30 minutes, mice were gently placed on a moving (4 rpm) rotarod for 30 seconds. Then, over a period of 235 seconds, rotarod speed was increased to 40 rpm. The trial ended if mice stopped walking for 2 revolutions or dropped onto the paddle. Mice were trained 3 times per day with a 30-minute interval between each training session over a period of 3 days. The following week, mice were tested on a single day using the training protocol.

#### Survival.

All deaths were recorded. Mice that lost greater than 20% maximal body weight were euthanized and recorded as deaths.

### Purkinje cell quantification

Quantification of Purkinje cells was performed as described previously (19). Briefly, midline sagittal sections were stained with calbindin to identify Purkinje cells. The number of cells was normalized to the length of the Purkinje layer as measured by NIH ImageJ software.

### Vinblastine treatment

Mice were injected with vinblastine (0.04 mg/g) i.p. at 7 weeks of age as previously described (37). Liver was collected 2 hours after injection and processed for immunofluorescence staining.

#### Statistics.

GraphPad Prism 7.0 was used to determine significance (*P* < 0.05), *F* (F-statistic) and *t* (T-statistic) values. Unpaired Student’s *t* test (2 tailed) and 1-way or 2-way ANOVA were used as indicated in the figure legends. A *P* value less than 0.05 was considered significant. All error bars are SEM.

#### Study approval.

All procedures involving mice were approved by the University of Michigan Committee on Use and Care of Animals (PRO00008133) and conducted in accordance with institutional and federal guidelines.

## Author contributions

EAL and APL conceptualized the study. EAL and MS provided methodology. EAL, MS, CM, and CC provided investigation. HP obtained resources. EAL and APL wrote the original draft. MS, CM, CC, and HP wrote, reviewed, and edited the manuscript. EAL, MS, and APL visualized data. APL supervised the study and acquired funding.

## Supplementary Material

supplemental data

## Version 1 09/15/2020

In-Press Preview

## Version 2 10/15/2020

Electronic publication
