# Supplementary material for: Fbxo2 mediates clearance of damaged lysosomes and modifies neurodegeneration in the Niemann-Pick C brain
Source: JCI Insight. 2020 Oct 15;5(20):e136676. doi: 10.1172/jci.insight.136676 (PMC7605537; doi:10.1172/jci.insight.136676)
Supplement: supplemental data [file jciinsight-5-136676-s175.pdf]

Supplemental Data

Fbxo2 mediates clearance of damaged lysosomes and modifies neurodegeneration in the Niemann-Pick C brain

Elaine A. Liu[1, 2, 3], Mark L. Schultz[1], Chisaki Mochida[4], Chan Chung[1], Henry L. Paulson[5], Andrew P. Lieberman[1]

[1]Department of Pathology, University of Michigan Medical School, Ann Arbor, MI 48104 USA

[2]Cellular and Molecular Biology Graduate Program, University of Michigan Medical School, Ann Arbor, MI 48109 USA

[3]Medical Scientist Training Program, University of Michigan Medical School, Ann Arbor, MI 48109 USA

[4]Yamaguchi University School of Medicine, Ube, Yamaguchi 755-8505 Japan

[5]Department of Neurology, University of Michigan Medical School, Ann Arbor, MI 48109 USA

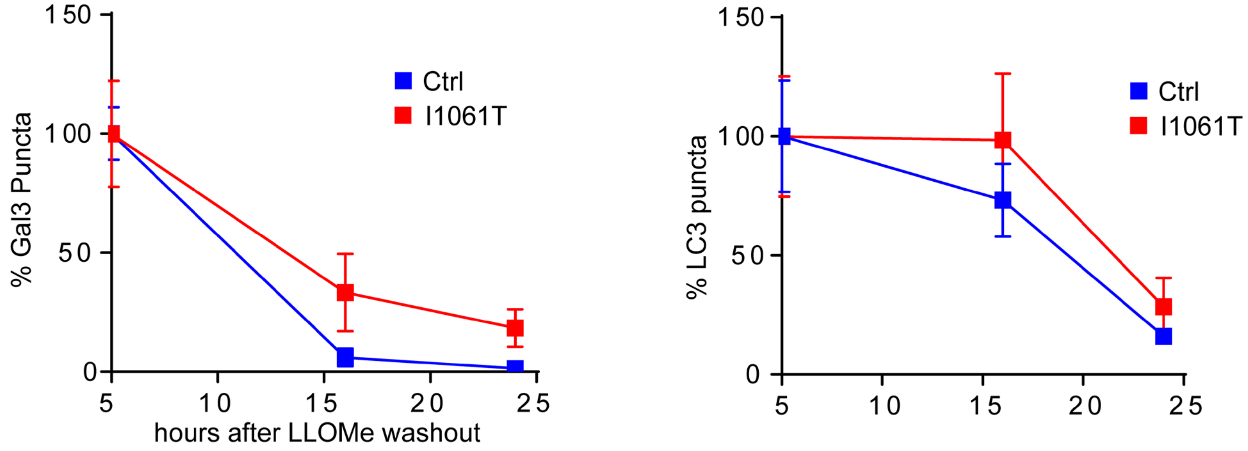

Supp. Fig. 1. I1061T patient fibroblasts exhibit similar rates of Gal3 and LC3 clearance
CTRL and I1061T patient fibroblasts were treated with Veh or 2mM LLOMe for 1hr and stained for Gal3 or LC3 at indicated times after washout. % of Gal3 or LC3 puncta clearance is plotted. Data are shown as mean ± s.e.m. from 3-4 independent experiments. Clearance of Gal3 and LC3 are not significantly different (P>0.5) by two-way ANOVA with Sidak’s test.

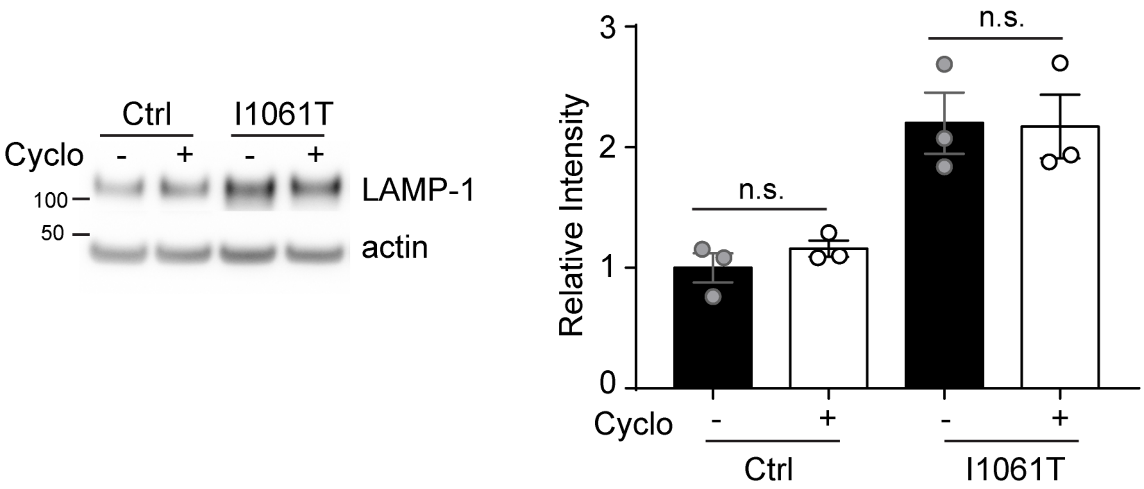

Supp. Fig. 2. LAMP-1 levels do not change after cyclodextrin treatment
CTRL and I1061T patient fibroblasts were treated with Veh or 1mM cyclodextrin (Cyclo) for 48 hrs, then treated with 2mM LLOMe for 1 hr. LAMP-1 levels were analyzed by western blot. Data are shown as mean ± s.e.m. from 3 independent experiments. n.s., not significant

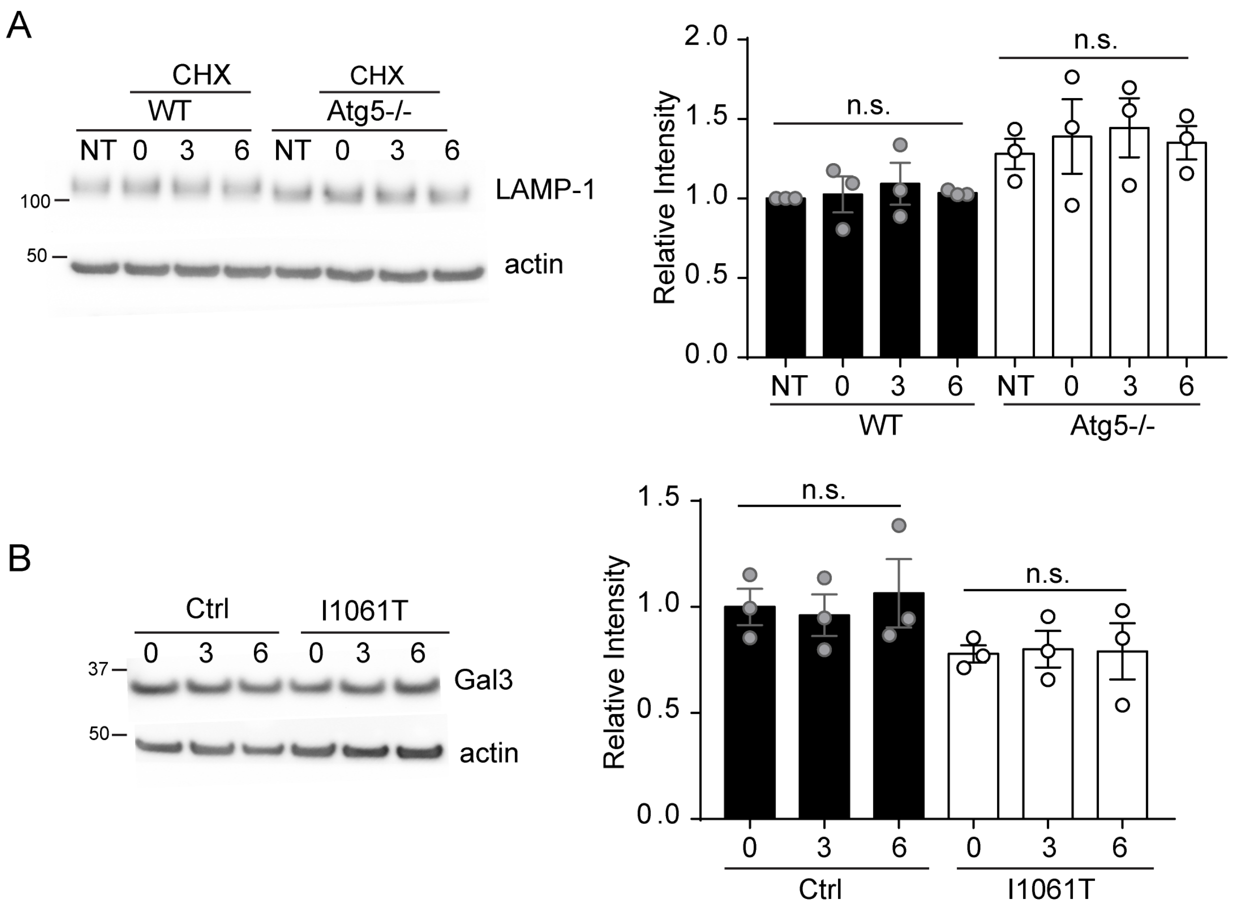

Supp. Fig. 3. LAMP-1 and Gal3 levels are not changed in the absence of LLOMe treatment

(A) WT and Atg5-/- MEFs were treated with 30μg/mL CHX for the indicated times and LAMP-1 levels were analyzed by western blot. (B) CTRL and I1061T patient fibroblasts were treated with CHX for the indicated times and Gal3 levels were analyzed by western blot. Data are shown as mean ± s.e.m. from 3 independent experiments. n.s., not significant

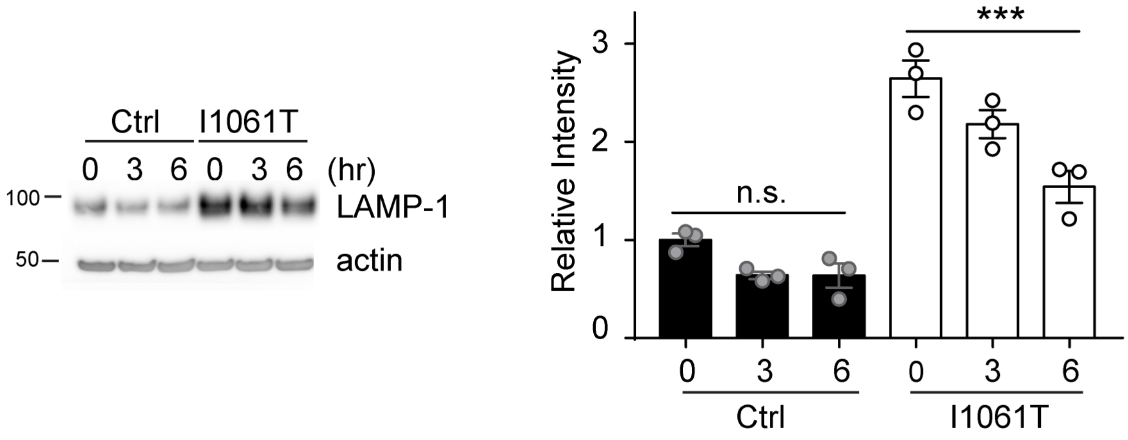

Supp. Fig. 4. LAMP-1 levels are decreased after LLOME treatment
CTRL and I1061T patient fibroblasts were treated with 30μg/mL CHX and 2mM LLOMe for 1hr. CHX treatment continued for the indicated time points. LAMP-1 levels were analyzed and quantified at the right. This blot was derived from the same gel as in Figure 4C and has the same loading control. Data are shown as mean ± s.e.m. from 3 independent experiments. n.s., not significant, ***$P \leq 0.001$ by one-way ANOVA $F=40.7$

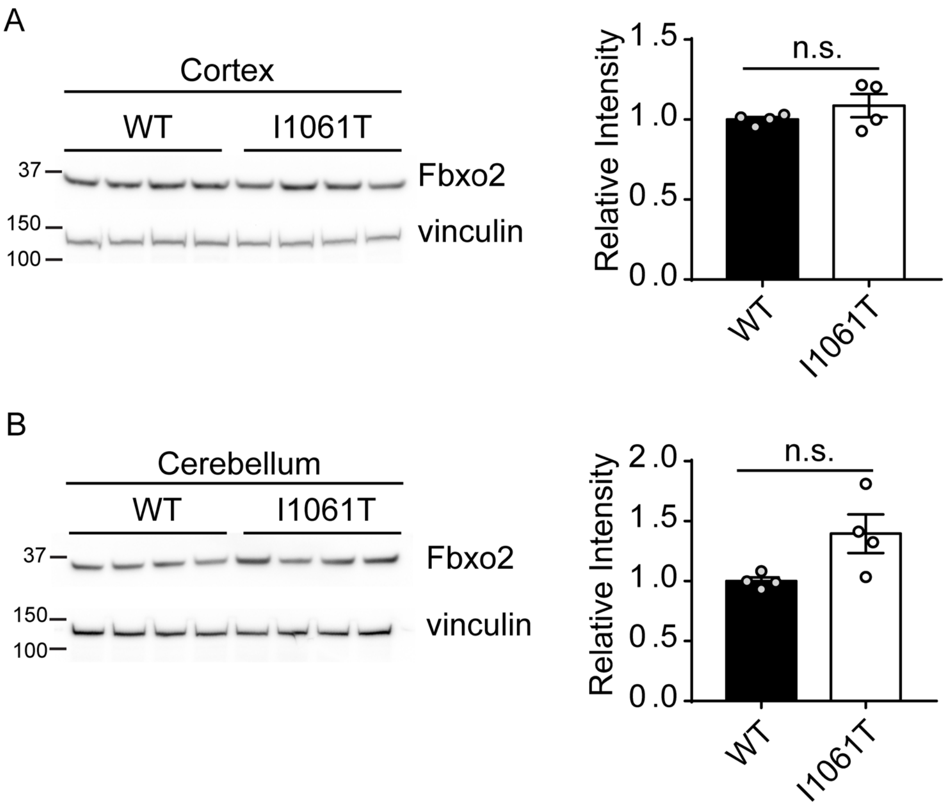

Supp. Fig. 5. WT and Npc1-I1061T mice have similar levels of Fbxo2
Cortex and cerebellum lysates were collected from 12wk WT and Npc1-I1061T mice. Fbxo2 levels are quantified at the right. N=4 mice per genotype. Data are shown as mean ± s.e.m. n.s., not significant by t-test.

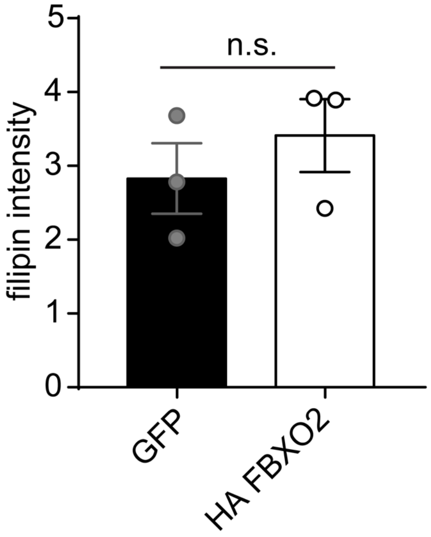

Supp. Fig. 6. Overexpression of HA FBXO2 does not affect cholesterol storage
I1061T patient fibroblasts were electroporated with HA FBXO2 and stained for filipin. Data are shown as mean ± s.e.m. from 3 independent experiments. Data are shown as mean ± s.e.m. n.s., not significant by t-test.

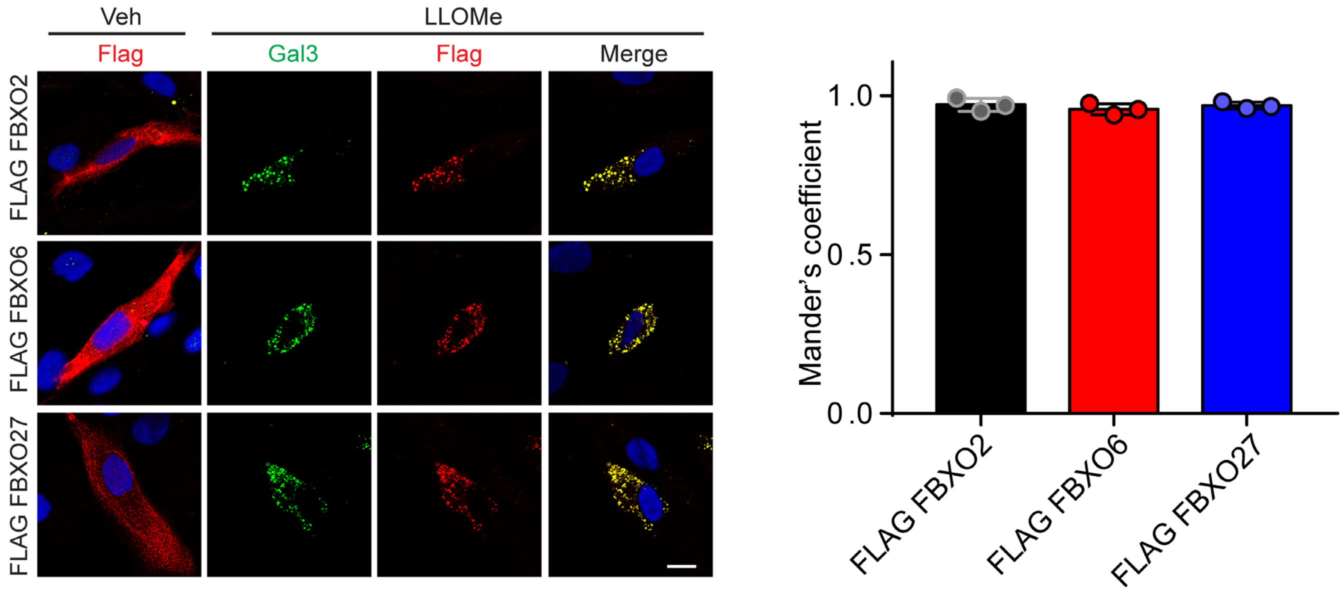

Supp. Fig. 7. FLAG FBXO2, FBXO6 and FBXO27 show similar levels of recruitment to damaged lysosomes

I1061T patient fibroblasts were transfected with FLAG constructs, treated with 2mM LLOMe for 1hr and stained 2hrs after washout. Mander's coefficients for co-localization of Gal3 and Flag are shown at the right. Data are shown as mean ± s.e.m. from 3 independent experiments. Scale bar: 25μm

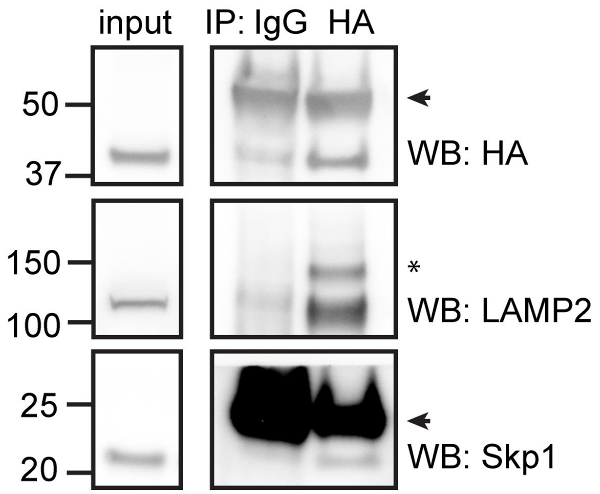

Supp. Fig. 8. Co-immunoprecipitation shows interaction of HA-FBXO2 with Skp1 and LAMP2

I1061T patient fibroblasts were electroporated with HA-FBXO2, and after 48hr treated with vehicle for 2hr. Lysates were immunoprecipitated with either HA antibody or control IgG. Arrowheads at ~50kD and ~25kD indicate immunoglobulin heavy and light chains, respectively. Asterisk denotes a non-specific band detected by the LAMP2 antibody.

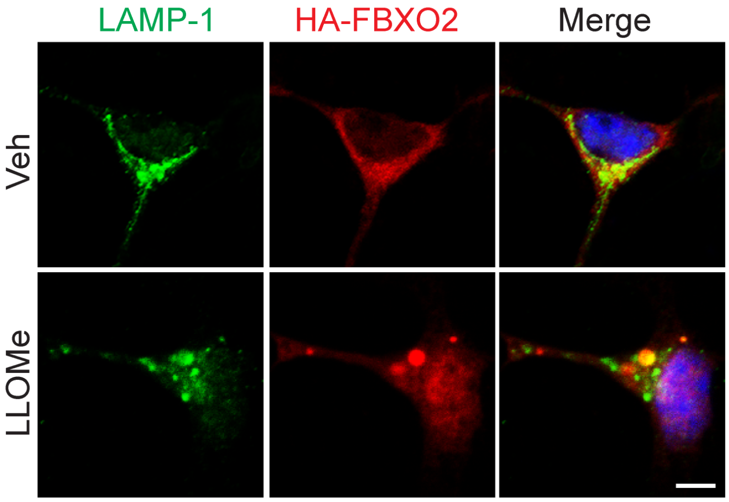

Supp. Fig. 9. FBXO2 is recruited to damaged lysosome in primary cortical neurons

WT primary cortical cultures were transfected with HA-FBXO2 and treated with vehicle (Veh) or 2mM LLOMe for 1hr and stained after 2hrs. Scale bar:10μm

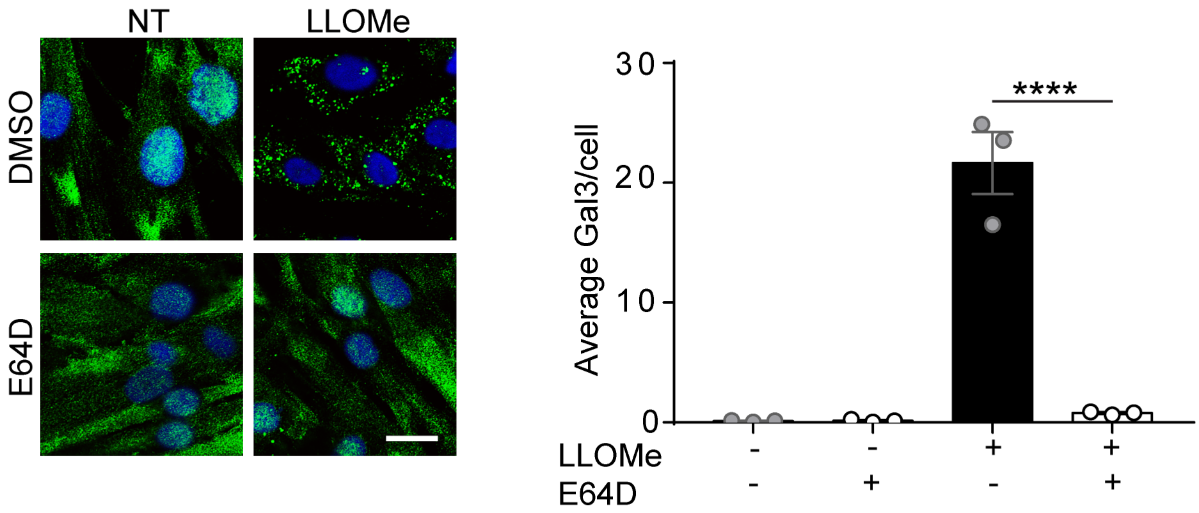

Supp. Fig. 10. Pre-treatment with E64D prevents lysosomal damage
I1061T patient fibroblasts were treated with 100μM E64D for 30min, then treated with 2mM LLOMe for 1 hr and stained for Gal3. Gal3 puncta per cell quantified at the right. Data are shown as mean ± s.e.m. from 3 independent experiments. ****$P \leq 0.0001$ by one-way ANOVA with Tukey's multiple comparisons. $F=67.39$ Scale bar: 25μm

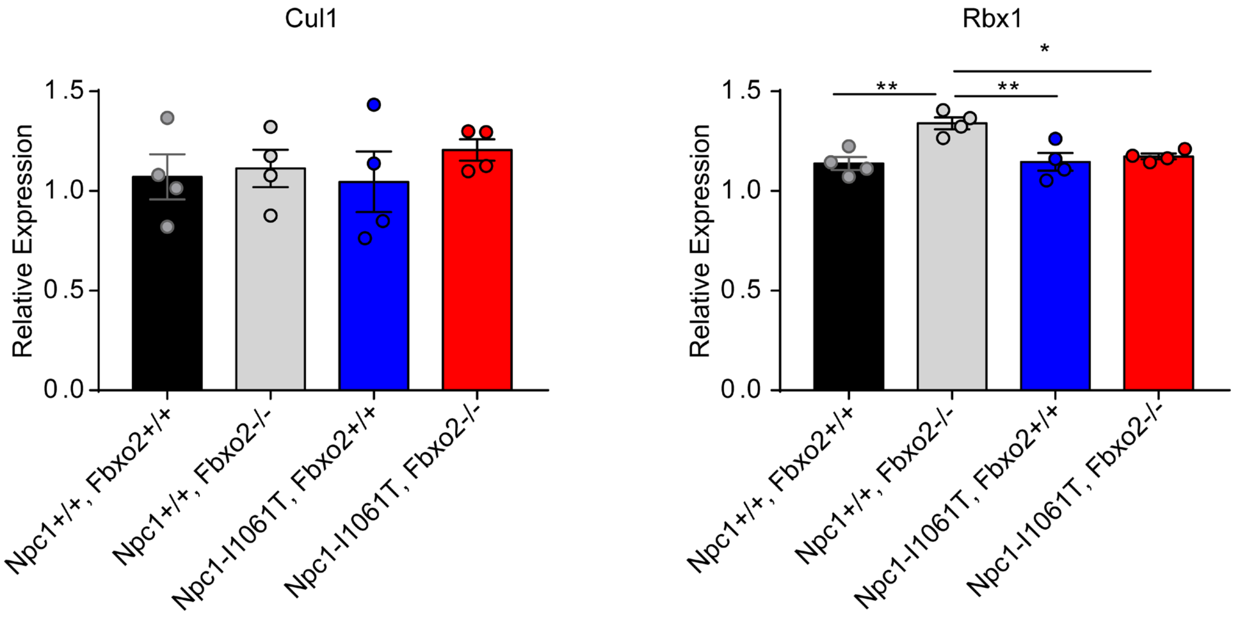

Supp. Fig. 11. Expression of SCF components in Fbxo2-/- mice
Relative expression of Cul1 and Rbx1 was determined by qPCR in 8wk brainstem. N=4 mice per genotype. Data are shown as mean ± s.e.m. *P ≤ 0.05, **P ≤ 0.01 by one-way ANOVA

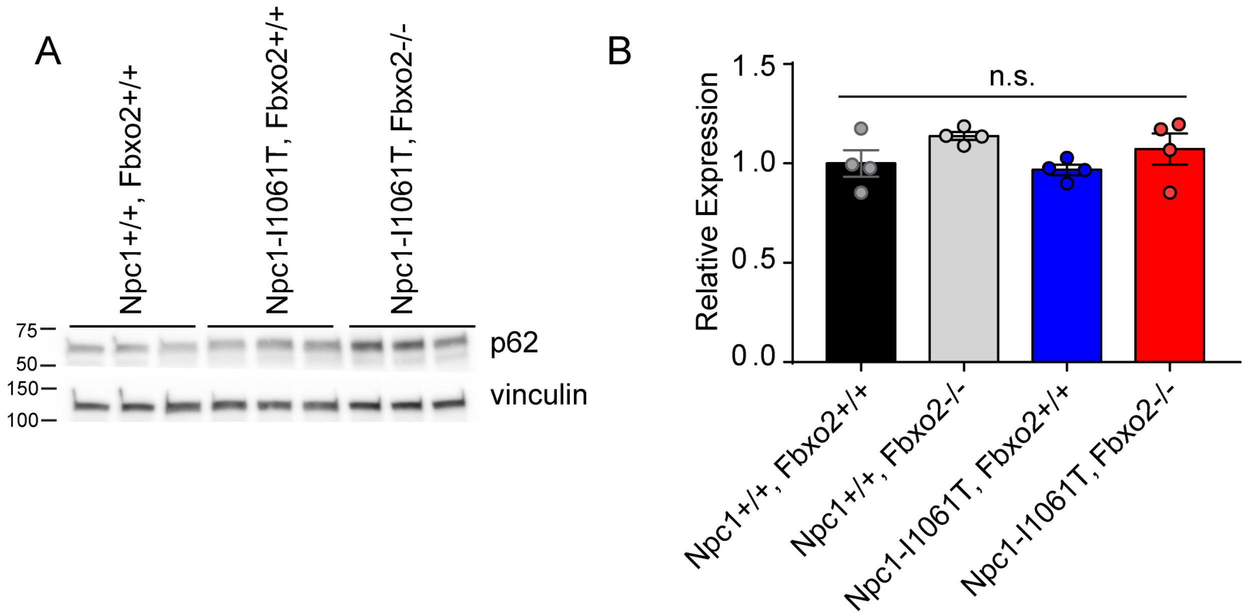

Supp. Fig. 12. p62 protein levels are increased in Npc1-I1061T, Fbxo2-/- mice without changes in gene expression
Brainstem was collected from 8 wk mice and p62 levels were analyzed by **(A)** western blot or **(B)** qPCR. N=4 mic per genotype. Data are shown as mean ± s.e.m. n.s., not significant by one-way ANOVA.
